# Eigenvalue Ratios Reveal Shared Binding Pocket Shapes in RNA and Protein Structures

**DOI:** 10.34133/csbj.0022

**Published:** 2026-04-20

**Authors:** Leïla Ziani, Anne Badel, Léa Dufay, Delphine Flatters, Leslie Regad, Anne-Claude Camproux

**Affiliations:** Université Paris Cité, CNRS UMR 8251 INSERM ERL U1133, Unité de Biologie Fonctionnelle et Adaptative (BFA), F-75013 Paris, France.

## Abstract

Molecular recognition in drug design relies on accurate characterization of ligand-binding pockets on macromolecular targets such as proteins and RNA. While protein binding sites have been extensively described, the geometric organization of RNA pockets remains comparatively underexplored. Here, we introduce a unified and size-independent geometric framework to describe and compare RNA and protein binding pocket shapes. Pocket geometry is captured using size-independent and residue-agnostic measures of global anisotropy, enabling direct comparison across RNA and protein binding pockets without introducing macromolecule-specific assumptions. This approach defines 4 interpretable pocket shape archetypes: sphere-like, rod-like, disk-like, and strongly anisotropic. Application to balanced datasets of 300 RNA and 300 protein binding pockets reveals a largely shared geometric landscape, with substantially overlapping shape descriptors within each archetype. However, archetype frequencies differ: Sphere-like pockets are more frequent in proteins, whereas disk-like and strongly anisotropic pockets are enriched in RNA, while rod-like pockets occur at comparable frequencies. Notably, strongly anisotropic pockets lacking a dominant symmetry axis represent a substantial fraction of pockets in both datasets. By organizing diverse binding sites into a small number of reproducible geometric regimes, this framework reduces structural heterogeneity and provides a transferable geometrical reference for comparative analysis of RNA and protein pocket architectures, thereby supporting the exploration of RNA pocket accessibility in structure-based studies.

## Graphical Abstract

**Figure:**
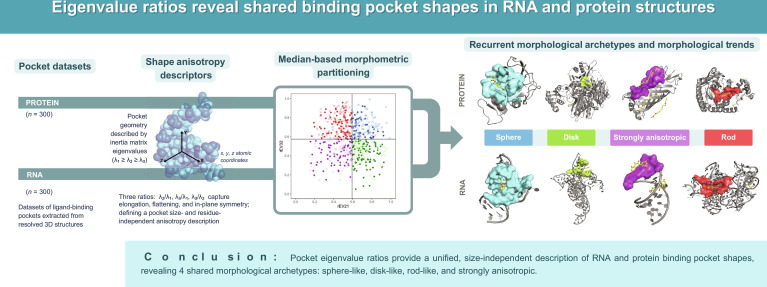

## Introduction

Binding pockets constitute the structural receptacles of ligands, and their shape governs recognition, specificity, and selectivity. While extensive structural data exist for proteins, our understanding of RNA pocket shape organization remains fragmented, despite the growing number of RNA–ligand complexes now available. Establishing a quantitative framework that allows pockets from RNA and proteins to be compared on equal morphometric basis is therefore a crucial step toward understanding the underlying principles of molecular recognition. To move beyond surface and composition-based metrics, eigenvalue-based descriptions derived from the inertia matrix provide a robust and size-independent framework for quantifying pocket shape across RNA and proteins. From a morphometric standpoint, a binding pocket can be represented as a 3-dimensional point cloud formed by the atomic coordinates of the residues or nucleotides that delimit the pocket. The inertia (or covariance) matrix of this point cloud summarizes how pocket atoms are spatially distributed around their centroid along the principal axes, providing a size-independent description of pocket shape. Traditional eigenvalue-based approaches, derived from this matrix, have been applied separately to RNA and protein structures by discretizing shape space into a small number of predefined shape categories (e.g., rod-, disk-, or sphere-like) based on threshold values of principal axis ratios, thereby assigning each pocket to the nearest idealized form. Such approaches used in protein studies [1,2], and in global RNA shape analyses [3] or pocket shape characterizations [4], describe molecular shapes through the principal moments of inertia.

However, these approaches typically rely on 2 principal axis ratios (*λ*_2_/*λ*_1_ and *λ*_3_/*λ*_1_), while the local anisotropy ratio (*λ*_3_/*λ*_2_), which captures symmetry within the minor plane, is less commonly considered. This additional ratio captures symmetry within the minor (y-z) plane and helps distinguish between different anisotropic shape organizations, such as strongly anisotropic and cylindrical shapes. Here, we extend this framework by integrating the complete set of eigenvalue ratios (*λ*_2_/*λ*_1_, *λ*_3_/*λ*_2_, and *λ*_3_/*λ*_1_).

More practically, we primarily rely the morphometric partitioning of pocket shapes on the 2 ratios *λ*_2_/*λ*_1_ and *λ*_3_/*λ*_2_, which capture global elongation and cross-sectional symmetry, respectively, while the third ratio, *λ*_3_/*λ*_1_, is reported as a derived descriptor of overall anisotropy. This formulation enables a size-independent decomposition of pocket anisotropy and supports a morphometrically guided partition of shape space, in line with principles established in geometric morphometrics [5]. These eigenvalue ratios are used within a morphometric decision tree that partitions the shape space into interpretable anisotropy archetypes, allowing RNA and protein pockets to be analyzed jointly.

In this study, we assembled 2 balanced datasets of 300 RNA and 300 protein ligand-binding pockets and applied a morphometric decision tree, based on eigenvalue ratios to characterize, and compare their shapes. This decision tree, based on global median thresholds derived directly from combined RNA and protein data, provides a robust and interpretable partitioning of the continuous pocket shape space into shared morphological archetypes, suitable for joint RNA–protein comparison. This framework defines 4 interpretable pocket archetypes: sphere-like, rod-like, disk-like, and strongly anisotropic, that provide a common reference for joint RNA–protein comparison. Importantly, this decision tree does not impose similarity between RNA and protein pockets but preserves the ability to detect systematic shape differences if present.

This analysis enables (a) the definition of interpretable and scale-independent pocket shape archetypes derived directly from the data, (b) the unified assignment of RNA and protein pockets within a common shape space, and (c) the comparative analysis of archetype distributions and morphometric characteristics across macromolecular types.

## Materials and Methods

### RNA– and protein–ligand complex datasets

We considered 2 independent datasets of 300 RNA binding pockets and 300 protein binding pockets extracted from RNA–small molecule complexes and protein–small molecule complexes, respectively.

Recent curated resources such as HARIBOSS [6] or NAKB [7], derived from the Protein Data Bank (PDB) [8], provide an opportunity to analyze RNA–ligand complexes at scale and to compare their binding pockets with those of proteins. In this study, RNA–small molecule complexes were obtained from the HARIBOSS database [6], which initially contains approximately 1,000 RNA–ligand structures. A multistep filtering procedure was applied to reduce structural and chemical biases. Complexes containing proteins or DNA, exceptionally large RNA assemblies (>2,000 nucleotides), and chemically modified nucleotides were excluded, resulting in 380 complexes. To reduce redundancy, identical RNA chains within the same PDB entry were identified based on sequence identity, and a single representative chain was retained for pocket analysis in multimeric assemblies. From the remaining entries, 254 distinct RNA–small molecule complexes with a maximal resolution of 4.5 Å were selected. Their RNA structural classes were annotated a posteriori to assess dataset composition. Riboswitches and structured functional RNAs (including ribozymes, transfer RNAs, aptamers, synthetic RNAs, and introns) account for 39% and 34.6% of the dataset, respectively, while ribosomal RNAs represent 16.5%, small regulatory RNAs 7.9%, and mRNAs 2%.

A protein binding pocket dataset was constructed from a curated internal repository of protein–small molecule complexes derived from the PDB using a standardized extraction pipeline.

Binding pocket selection proceeded through several controlled steps to ensure structural quality and diversity. First, structural quality filtering was applied to the source protein–ligand complexes, retaining only structures with a resolution ≤4.5 Å. Second, redundancy was limited by retaining a single binding pocket per protein chain associated with a single ligand. Third, a total of 300 protein pockets were selected through 3 complementary criteria: (a) promoting diversity across homologous superfamilies using the CATH classification system, which organizes protein domains hierarchically into Class, Architecture, Topology, and Homologous superfamily levels; (b) enforcing a unique UniProt identifier for each selected protein; and (c) ensuring ligand uniqueness across selected complexes. The final dataset spans more than 250 distinct CATH superfamilies (level 4), ensuring broad structural diversity of protein binding pockets. The resulting dataset therefore reflects a controlled selection from a curated PDB-derived repository rather than a direct random sampling from the PDB. The complete list of selected PDB identifiers, chains, ligands, UniProt identifiers, and CATH annotations is provided in Table S1 to ensure full reproducibility.

The average resolution of the protein dataset (2.27 Å) is comparable to that of the RNA dataset (2.65 Å). More importantly, pocket selection was based exclusively on structural criteria, and ligand identity and chemistry were not used as selection filters. RNA and protein complexes were selected independently to avoid confounding pocket geometry with ligand properties. Consequently, the composition of the 2 datasets was not constrained by ligand overlap, and pocket geometry reflects the structural organization of the macromolecular environment rather than ligand-driven bias.

### Pocket definition and preprocessing

#### Structure preprocessing and coordinate harmonization

**Fig. 1.**
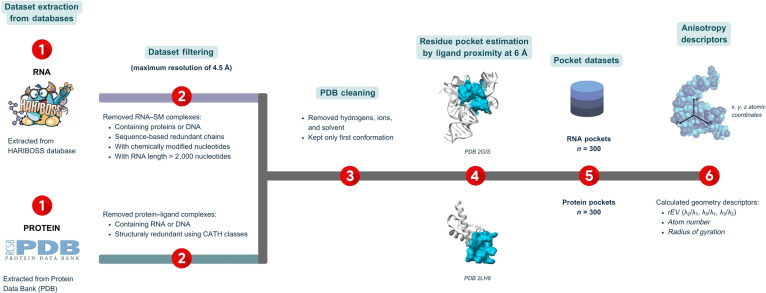
Overview of RNA and protein pocket dataset construction and morphometric characterization. (1) Structural data were collected from curated RNA–ligand and protein–ligand complex databases and subjected to (2) successive filtering to remove redundant entries, mixed macromolecular complexes, and low-resolution structures. (3) Protein Data Bank (PDB) files were cleaned by removing solvent molecules, ions, and hydrogen atoms. (4) Ligand-centered pocket estimation was then performed using a unified geometric protocol, resulting (5) in 2 balanced datasets of 300 RNA and 300 protein binding pockets. (6) For each pocket, morphometric descriptors were computed, including inertia-matrix eigenvalue ratios quantifying pocket anisotropy (*rEV21*, *rEV32*, and *rEV31*) and size-related descriptors such as the number of atoms and the radius of gyration.

All RNA and protein structures were processed using a standardized preprocessing pipeline prior to pocket extraction (Fig. 1). Solvent molecules and ions were removed, and all analyses were performed exclusively on heavy atoms. Because a subset of deposited structures contained explicit hydrogen atoms, all hydrogen atoms were systematically removed prior to pocket extraction in order to homogenize structural representations and ensure consistent geometric descriptions across datasets. When multiple atomic conformations were present in PDB files, only the first listed conformation was retained consistently for both macromolecules and ligands.

#### Pocket definition

##### Residue-level versus atom-level pocket definition

Binding pockets were defined at the residue (or nucleotide) level rather than at the atom level. This choice was motivated by geometric and representational considerations. Atom-level pocket definitions can produce fragmented and spatially disconnected point clouds, particularly in RNA structures where ligand recognition often involves multiple noncontiguous nucleotides. Such fragmentation may compromise the stability of global shape descriptors derived from the spatial distribution of pocket atoms.

In contrast, residue-level definitions tend to preserve more the 3-dimensional continuity of binding sites by including complete contacting residues or nucleotides, yielding compact and coherent pocket representations that are better suited for global morphometric analysis. This representation also better maintains structural features such as helices, grooves, junctions, and backbone organization, which are essential for capturing the overall geometry of RNA and protein binding pockets. A representative comparison between atom-level and residue-level pocket definitions illustrates how atom-level estimation can produce fragmented and discontinuous point clouds, as exemplified by an RNA pocket defined at the atom level (Section S2). Such residue-based definitions are widely used in structural bioinformatics for protein binding sites and RNA–ligand complexes and preserve the structural and geometric integrity of binding regions [9–11].

##### Pocket estimation by ligand proximity

Both RNA and protein pockets were estimated based on proximity to the ligand, using respectively a Python script for RNA and PockDrug [12,13] for proteins. Binding pockets were defined as the set of complete residues having at least 1 heavy atom within 6 Å of any ligand heavy atom. While no consensus distance cutoff is universally established for RNA binding pockets, thresholds in the range of 4 to 6 Å are commonly used in protein–ligand pocket definitions and may be extended as an operational choice for RNA due to similar geometric considerations [9,14]. The 6-Å cutoff represents a compromise that captures the immediate interaction shell while preserving the global geometry of the binding site.

#### Control of ligand-driven and redundancy-related biases

##### Control of ligand-driven biases

Ligand identity, size, and chemistry were not used as selection criteria but were evaluated a posteriori to assess potential ligand-driven biases. RNA and protein–ligand complexes were selected independently under structural and resolution constraints to avoid artificially conditioning pocket geometry on ligand properties. Ligand chemical diversity was characterized using 2-dimensional Morgan fingerprints (radius = 2, 2,048 bits), and chemical space overlap was visualized using multidimensional scaling (Section S3). Ligand size distributions were also examined. These analyses were used exclusively as methodological controls.

##### Control of pocket redundancy-related biases

Each protein pocket corresponds to a unique macromolecular structure. Although a limited number of RNA complexes contained more than 1 pocket associated with the same ligand, potential nonindependence effects were explicitly evaluated. RNA and protein pocket shape comparisons were repeated after strict redundancy filtering (1 pocket per PDB–ligand–chain combination) to assess the potential impact of intra-PDB dependence (Section S4).

### Pocket anisotropy characterization

#### Pocket representation and eigenvalue-based shape descriptors

Each binding pocket was represented as a 3-dimensional point cloud defined by the atomic coordinates of all residues delimiting the pocket. Only heavy atoms were considered, following the preprocessing procedure described above. Atomic coordinates were centered prior to inertia matrix calculation. All atoms were treated uniformly, without atom-type or mass weighting, to preserve a purely geometric description of pocket shape.

Pocket geometry was characterized using the inertia (second-moment) matrix computed from centered atomic coordinates around the pocket centroid, which provides a purely geometric description of spatial dispersion. The 3 eigenvalues of this matrix denoted *λ*_1_, *λ*_2_, and *λ*_3_ and ordered by convention as *λ*_1_ ≥ *λ*_2_ ≥ *λ*_3_ quantify how pocket atoms are distributed along the 3 principal axes of inertia. *λ*_1_ reflects dispersion along the major axis, *λ*_2_ along the intermediate axis, and *λ*_3_ along the minor axis. The relative magnitudes of these eigenvalues capture the effective dimensionality of the pocket, ranging from elongated (1 dominant axis) to isotropic (similar dispersion along all axes). Eigenvalue-based descriptors of shape anisotropy are widely used in geometric morphometrics and multivariate shape analysis [5,15].

Because raw eigenvalues depend on pocket size, shape characterization was based on eigenvalue ratios, which are scale-invariant and bounded between 0 and 1. We considered 3 ratios: *rEV21* = *λ*_2_/*λ*_1_, *rEV32* = *λ*_3_/*λ*_2_, and *rEV31* = *λ*_3_/*λ*_1_. In this framework, *rEV21* describes global elongation, *rEV32* quantifies symmetry within the minor (y-z) plane, and *rEV31* provides a derived measure of overall anisotropy (Section S5). Because eigenvalue ratios range between 0 (extreme anisotropy) and 1 (perfect isotropy), median values provide a natural reference to position typical pocket geometries along a continuum from near-isotropic to moderately anisotropic shapes.

#### Size-related pocket descriptors

In parallel with shape characterization, pocket size was described using 2 descriptors: the number of atoms forming the pocket and the radius of gyration.

The radius of gyration was computed from the inertia matrix eigenvalues as:

$$R_{g}=\sqrt{\lambda_{1}+\lambda_{2}+\lambda_{3}}$$ (1)

providing a scalar measure of global spatial extent of pocket atoms around their center of mass, derived from the same geometric framework as the shape ratios.

Shape descriptors quantify pocket anisotropy independently of absolute size, whereas size descriptors capture volumetric properties. These 2 families of descriptors were therefore treated separately throughout the analysis to disentangle genuine geometric effects from size-related variations.

#### Robustness of shape descriptors to pocket definition

To ensure that the observed pocket shape trends are not artifacts of a specific pocket definition, we performed a dedicated sensitivity analysis to assess the robustness of inertia-based shape and size descriptors to variations in pocket estimation parameters. Two complementary analyses were conducted. First, shape and size descriptors were compared between atom-level and residue-level pocket definitions using a 6-Å ligand-centered cutoff. Second, residue-level pockets were estimated using cutoff distances of 4, 5, and 6 Å. These analyses were designed to evaluate methodological robustness rather than to select or optimize a specific pocket definition. All results are reported in Section S6.

### Morphometric decision scheme for archetypes

To obtain an interpretable discretization of pocket shapes, pockets were embedded in a 2-dimensional morphospace defined by (*rEV21*, *rEV32*). We adopted a morphometrically guided decision tree based on the algebraic interpretation of eigenvalue hierarchies. The ratio *rEV31* is algebraically dependent on *rEV21* and *rEV32* (*rEV31* = *rEV21* × *rEV32*). It was not used for archetype assignment but was reported as a derived descriptor of overall anisotropy within each archetype.

**Fig. 2.**
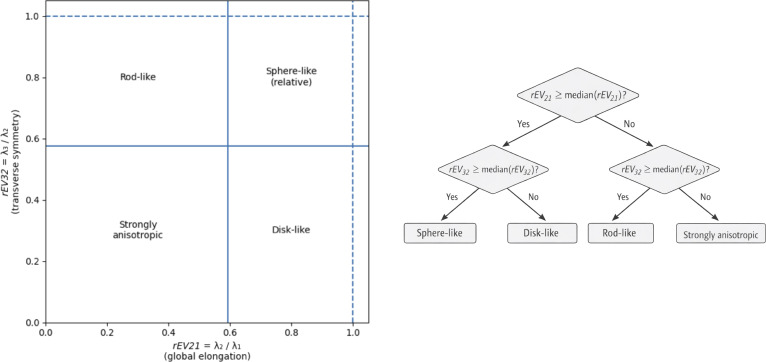
Median-based decision tree for morphometric classification of pocket shapes. Pocket shapes are classified using a median-based decision tree defined in the 2-dimensional eigenvalue-ratio space (*rEV21*, *rEV32*). Global dataset medians of *rEV21* and *rEV32* are used as splitting thresholds, partitioning the continuous shape space into 4 recurrent morphological archetypes: rod-like, disk-like, sphere-like, and strongly anisotropic pockets. The left panel shows the projection of pockets in the (*rEV21*, *rEV32*) plane with median-based boundaries (solid lines for medians and dotted lines for upper limits), while the right panel illustrates the corresponding decision tree used to assign each pocket to a shape archetype.

Partitioning thresholds were defined using the global medians of *rEV21* and *rEV32* computed on the combined RNA–protein dataset, ensuring a common reference frame, and limiting sensitivity to outliers. Four morphological archetypes were defined as illustrated in Fig. 2. This median-based decision tree partitions the continuous shape space into 4 recurrent morphological archetypes: sphere-like, rod-like, disk-like, and strongly anisotropic pockets.

Because binding pockets correspond to concave, solvent-exposed regions rather than closed solids, absolute isotropy thresholds are poorly suited to pocket geometry. The median-based, data-adaptive decision scheme therefore provides a robust and interpretable partitioning of pocket shapes suitable for joint RNA–protein comparison.

While larger datasets may slightly shift median values, the use of medians and a continuous decision framework ensure robustness to sampling variability, with potential uncertainty limited to pockets located near class boundaries. To explicitly assess the stability of this median-based partitioning with respect to sampling variability, we performed stratified bootstrap resampling of the RNA and protein datasets. For each of 1,000 bootstrap replicates generated independently within the RNA and protein sets, median ratio values and the resulting archetype assignments were recomputed. Partition stability was quantified using the variability of decision thresholds, the proportion of reclassified pockets, and the adjusted Rand index (ARI) relative to the reference classification.

We therefore adopt a data-adaptive, algebraic decision scheme yielding 4 stable and interpretable shape categories (archetypes) suitable for joint RNA–protein comparisons.

### Comparison of RNA and protein pocket shapes and archetypes

#### Shape descriptor comparison of RNA and protein pockets

For each of the 300 RNA and 300 protein binding pockets, 3 eigenvalue-ratio descriptors were computed from the inertia matrix of pocket atomic coordinates to describe the pocket shape. In addition, pocket size was characterized by 2 descriptors: the number of atoms and the radius of gyration.

Descriptive statistics, including means, SDs, and medians, were calculated for all descriptors separately on RNA, protein, and combined RNA–protein datasets. Comparisons between RNA and protein pocket ratios and pocket sizes were performed using Welch’s *t* tests [16], which account for unequal variances between groups. Multiple testing corrections using the Holm procedure were applied separately for shape descriptors (*rEV21*, *rEV32*, and *rEV31*) and size descriptors (number of atoms and radius of gyration). Given the large sample size (*n* = 300 per group), statistical significance was systematically interpreted in conjunction with standardized effect sizes (Cohen’s *d*) [17].

#### Continuous morphometric distances between pockets

While pocket shape archetypes were defined using a 2-dimensional decision tree based on *rEV21* and *rEV32*, continuous morphometric distances were computed to quantify shape similarity between RNA and protein pocket pairs and within and across archetypes.

Morphometric similarity between pockets was quantified in the 3-dimensional ratio space defined by (*rEV21*, *rEV32*, *rEV31*). Although *rEV31* is algebraically dependent on *rEV21* and *rEV32*, its explicit inclusion enables overall anisotropy to be represented continuously within a single metric space. Unweighted Euclidean distances between ratio vectors were used to quantify shape proximity between pockets. Small distances indicate highly similar anisotropy profiles, whereas larger distances reflect increasingly divergent elongation and cross-sectional symmetry properties.

To further characterize morphometric variability, we quantified both intra- and inter-archetype distances. For a given archetype, the intra-archetype distance was defined as the mean unweighted Euclidean distance between all pairs of pockets belonging to that archetype. Interarchetype distances were computed analogously using all pocket pairs formed between 2 distinct archetypes. Using pairwise distances rather than centroid-based measures allows the full distribution of morphometric variability to be captured without assuming homogeneous cluster structure. Distances were computed separately for RNA pocket, protein pocket, and RNA–protein pocket pairs.

#### Multivariate comparison of RNA and protein pocket shapes within archetypes

To assess whether RNA and protein pockets differ systematically within corresponding shape archetypes, a 1-factor multivariate analysis of variance (MANOVA) was performed separately for each archetype, with macromolecular types (RNA versus protein) as the explanatory factor.

The MANOVA was applied to the 2 independent eigenvalue ratios *rEV21* and *rEV32*. The third ratio, *rEV31*, was excluded from the multivariate model because it is algebraically dependent on *rEV21* and *rEV32* and was instead analyzed separately as a derived univariate descriptor of overall anisotropy. Pillai’s trace was used as the primary multivariate test statistic due to its robustness in balanced designs. Correlation structure and covariance homogeneity were assessed to ensure the suitability of the data for multivariate analysis. Archetype-specific MANOVA was used to test whether systematic RNA–protein shape differences remain after controlling for global pocket geometry. When relevant, complementary univariate comparisons were performed using Welch’s *t* tests with false discovery rate correction and systematic reporting of effect sizes (Cohen’s *d*), which were used as the primary measure of practical relevance.

Size-related descriptors (number of atoms and radius of gyration) were compared between RNA and protein pockets within each archetype using Welch’s *t* tests, with multiple testing correction applied across size descriptors and systematic reporting of effect sizes. Given the strong correlation between these 2 descriptors in both RNA and protein pockets, no multivariate testing was applied to size-related descriptors.

#### Differential occupancy of pocket shape archetypes in RNA and protein datasets

Continuous shape ratio descriptors were subsequently used to assign pockets to discrete morphological archetypes, using the median-based decision tree defined above, applied to the combined RNA–protein dataset. This geometric partitioning does not enforce equal archetype sizes and enables assessment of whether global RNA–protein differences arise from differential occupations of shared morphometric regimes rather than from systematic shape shifts within individual archetypes.

Following application of the median-based decision tree, the distribution of the 4 pocket shape archetypes was examined separately for RNA and protein datasets, as well as for the combined dataset. Archetype frequencies were reported as relative proportions.

Because the decision tree is median-anchored and does not enforce equal class sizes, differences in archetype frequencies directly capture how RNA and protein binding pockets populate the shared morphometric landscape. Archetype frequencies were compared using a chi-square test of independence, complemented by visual inspection using stacked bar plots and donut representations.

## Results

### Dataset controls and independence assessment

As the currently available RNA–ligand structural datasets are smaller and less chemically diverse than protein–ligand complexes, we performed a series of control analyses to identify and address potential sources of bias prior to comparing RNA and protein pocket shapes.

Ligand chemical space overlap (Section S3) and molecular weight distributions were evaluated. Despite minimal direct ligand overlap between RNA and protein datasets (8 shared ligands), ligands occupy overlapping regions of chemical space. This limited overlap was intentionally allowed to avoid conditioning pocket geometry on identical ligand chemotypes, as described in Materials and Methods. Ligand molecular weight distributions show substantial overlap, resulting in a small effect size (Cohen’s *d* = 0.26), although ligands binding RNA pockets exhibit a slightly higher average molecular weight than protein ligands (389.4 ± 175.0 Da versus 352.2 ± 124.3 Da). Overall, ligands from the 2 datasets occupy reasonably overlapping regions of chemical space, limiting the likelihood that observed pocket shape differences are primarily driven by ligand size or chemistry rather than intrinsic pocket geometry.

The sensitivity of pocket shape descriptors to alternative pocket definitions was further evaluated, including atom-level versus residue-level representations and residue-based cutoff distances (4 to 6 Å) (Section S6). While residue-level definitions increase absolute pocket size descriptors, this effect primarily impacts pocket extent rather than relative shape organization. In contrast, atom-level definitions lead to fragmented and discontinuous point clouds, particularly in RNA complexes, resulting in unstable geometric representations. Overall, shape descriptors remain highly stable across definitions, whereas size descriptors vary as expected with pocket expansion. Together, these results support the robustness of inertia-based shape descriptors to pocket definition and motivate the use of residue-level representations in subsequent analyses.

In addition, because a subset of deposited structures contained explicit hydrogen atoms, we evaluated the sensitivity of inertia-derived pocket descriptors to hydrogen inclusion (Section S7). This analysis was performed on a subset of complexes available both with and without explicit hydrogen atoms (20 RNA pockets and 11 protein pockets). Pockets were independently estimated for both structural representations using the same proximity-based procedure. In practice, the resulting pockets corresponded to the same set of pocket-defining residues, with the only difference being the presence of hydrogen atoms in the atomic representation. Eigenvalue ratios remained highly correlated between no-H and with-H representations across RNA and protein pockets (*r* > 0.96 for *rEV21*, *rEV32*, and *rEV31*). Although hydrogen inclusion increased the number of pocket atoms, changes in eigenvalue ratios were not associated with changes in atom count (|*r*| ≤ 0.18). Together, these results indicate that inertia-based shape descriptors are robust to hydrogen inclusion when the same residue-defined pocket membership is preserved.

Potential dependence between pockets originating from the same macromolecular context was also examined. While all protein pockets originate from distinct PDB entries, a limited subset of RNA PDB structures contributes more than 1 pocket associated with the same ligand, reflecting the modular organization of some RNA–ligand complexes and the currently more limited structural diversity of available RNA structures. Pairwise morphometric distances between such pockets are comparable to those observed between unrelated pockets in the overall distribution, with most values falling within the central range of the global distance distribution. This suggests that pockets extracted from the same macromolecular context are not systematically near-duplicate geometries. To further control for potential residual dependence in global RNA–protein comparisons, we repeated statistical RNA–protein pocket shape comparisons after retaining a single representative RNA pocket per PDB–ligand–chain combination. This conservative filtering yielded highly similar RNA–protein median values, archetype frequencies, and effect sizes, indicating that the main comparative results are robust to potential residual intra-PDB dependence (Section S4).

Finally, the stability of the median-based morphometric framework itself was assessed using bootstrap resampling of the RNA and protein datasets, as detailed later in Results. We next compared RNA and protein binding pockets using morphometric shape descriptors.

### Global pocket shape landscape and median-based descriptors

To characterize the global morphometric landscape of ligand-binding pockets, we first analyzed the distribution of eigenvalue ratios and size-related descriptors computed on the separate and combined RNA and protein datasets (Table 1). For RNA–protein comparisons, *P* values were adjusted for multiple testing using Holm’s method, applied separately to shape descriptors (eigenvalue ratios) and to size-related descriptors (number of atoms and radius of gyration).

**Table 1.** Comparison of RNA and protein binding pocket descriptors. Mean values (± SD) are reported for RNA and protein pockets. Differences between macromolecular types were assessed using Welch’s *t* tests. *P* values were adjusted for multiple testing using Holm’s procedure, applied separately to shape descriptors (eigenvalue ratios *rEV21*, *rEV32*, and *rEV31*) and to size-related descriptors (number of atoms and radius of gyration). Standardized effect sizes (Cohen’s *d*) are reported alongside adjusted *P* values to quantify the magnitude of observed differences. Global medians are also indicated.

Descriptor	RNA mean (SD)	Protein mean (SD)	*P* value (Holm)	Cohen’s *d*	RNA + Protein median
*rEV21*	0.572 (0.171)	0.600 (0.165)	4.437e−02	−0.165	0.592
*rEV32*	0.547 (0.184)	0.600 (0.182)	8.802e−04	−0.289	0.575
*rEV31*	0.309 (0.128)	0.360 (0.144)	1.916e−05	−0.372	0.328
*Number of atoms*	244.713 (91.756)	176.353 (57.169)	7.175e−25	0.894	200.500
*Radius of gyration*	9.248 (1.304)	8.759 (1.062)	6.222e−07	0.412	9.019

The eigenvalue ratios provide scale-invariant descriptors of pocket anisotropy, reflecting the relative spatial distribution of pocket atoms along the principal inertia axes rather than scale-dependent properties such as volume, radius of gyration, or atom count.

Comparative analysis shows that all 3 eigenvalue ratios (*rEV21*, *rEV32*, and *rEV31*) differ significantly between RNA and protein pockets (Table 1). However, these differences remain limited in magnitude, with effect sizes ranging from very small for *rEV21* to small-to-moderate for the secondary ratios. Notably, *rEV21* exhibits the smallest effect size, indicating that the primary elongation of binding pockets is largely conserved between RNA and protein systems.

Projection of all RNA and protein pockets onto the (*rEV21*, *rEV32*) plane reveals a continuous and structured shape landscape (Fig. 2), characterized by extensive overlap between the 2 macromolecular types. RNA- and protein-specific medians lie close to the global median, indicating subtle population-level shifts rather than distinct or separable geometric regimes. This observation supports the use of a common, dataset-wide median-based reference framework for describing the global pocket shape landscape.

Across the pooled RNA–protein dataset, global median eigenvalue ratios (mrEV21 ≈ 0.59; mrEV32 ≈ 0.57; mrEV31 ≈ 0.33; Table 1) indicate that most binding pockets exhibit moderate anisotropy rather than near-isotropic geometries. Median *rEV21* values around 0.6 correspond to shapes that remain relatively close to isotropy while displaying a modest preferential extension along the primary inertia axis, rather than a near-spherical organization.

In contrast to the modest shifts observed for eigenvalue ratios, pocket size descriptors reveal a markedly different pattern. Pocket size was described by the number of atoms forming each pocket and the radius of gyration. RNA pockets contain on average more than 60 additional atoms compared to protein pockets (Welch’s *t* test, *P* ≪ 0.001), with a large effect size (Cohen’s *d* > 0.8). Consistently, the radius of gyration differs significantly between RNA and protein pockets (*P* ≪ 0.001) with a moderate effect size (Cohen’s *d* ≈ 0.41), indicating a more extended spatial distribution of RNA pocket atoms around their center of mass than protein ones.

Together, these results indicate that RNA binding pockets are systematically larger in terms of atomic composition, while differences in spatial extension are more moderate, and shape anisotropy remains broadly conserved across macromolecular types.

### Morphological archetypes define the dominant organization of the shape space

#### Definition of morphological archetypes

To structure the continuous pocket shape space into interpretable morphological archetypes, we relied on 2 complementary eigenvalue ratios, *rEV21* and *rEV32*, which jointly capture the primary elongation and secondary anisotropy. Using the global medians of the combined RNA–protein dataset as reference values, this space was partitioned into 4 morphological archetypes: sphere-like (high *rEV21* and *rEV32*), rod-like (high *rEV32* and lower *rEV21*), disk-like (high *rEV21* and lower *rEV32*), and strongly anisotropic (low *rEV21* and *rEV32*) (Fig. 2).

This interpretation remains anchored to the normalized [0,1] scale of the eigenvalue ratios: Values close to 1 correspond to quasi-isotropic or weakly anisotropic shapes, intermediate values (~0.5 to 0.7) to moderate anisotropy, and low values (<0.4) to pronounced, though not necessarily extreme, anisotropy. The use of global medians provides an empirical reference to position biologically observed pocket geometries within the occupied morphometric space. This median-based partitioning provides a relative, data-adaptive, and scale-independent definition of archetypes, avoiding fixed or idealized geometric thresholds that are poorly suited to the continuous nature of binding pocket shapes.

The robustness of this archetype definition was assessed using stratified bootstrap resampling. Stability analyses revealed a high concordance with the reference partition, with a median ARI of 0.92 and a 95% bootstrap interval ranging from 0.82 to 0.98. Across bootstrap replicates, 68.5% of partitions exhibited an ARI ≥ 0.90 and 99.1% an ARI ≥ 0.80, while the median proportion of reclassified pockets remained low (3%). Together, these results indicate that the median-based morphometric framework is largely insensitive to sampling-induced variability.

Importantly, size-related descriptors do not drive archetype assignment. Radius-of-gyration distributions show substantial overlap across archetypes, indicating that the median-based morphometric classification primarily reflects shape anisotropy rather than pocket size (Section S6).

**Fig. 3.**
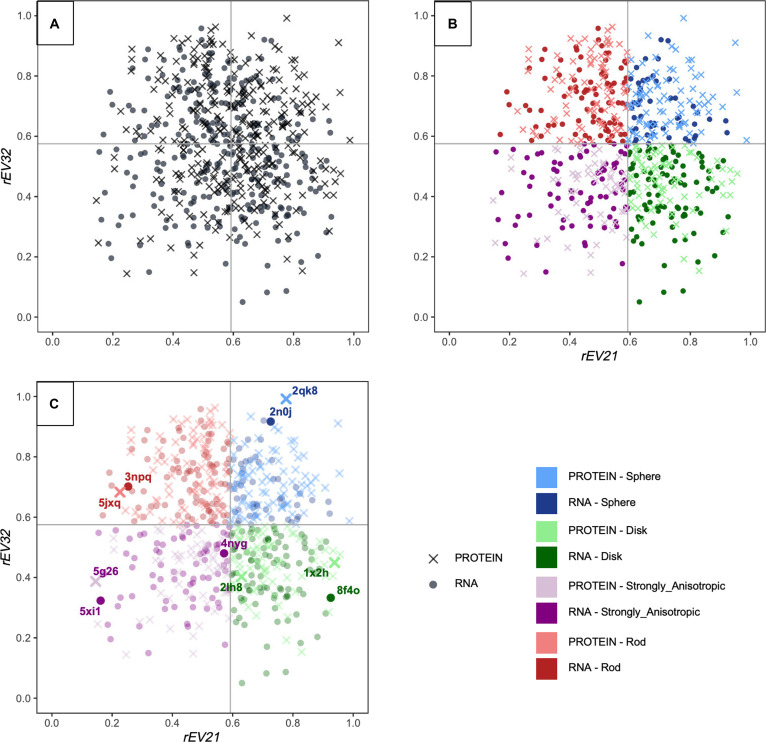
Global morphometric landscape and archetypal partitioning of RNA and protein binding pockets. (A) Global morphometric landscape of RNA and protein binding pockets. Projection of RNA and protein binding pockets onto the shape space defined by the 2 eigenvalue ratios *rEV21* (*λ*_2_/*λ*_1_, global elongation) and *rEV32* (*λ*_3_/*λ*_2_, cross-sectional symmetry). Each point represents a single binding pocket, with symbols indicating the macromolecular type. Light gray lines indicate the global medians computed on the combined RNA–protein dataset and serve as reference values. RNA and protein pockets largely overlap in this continuous space, highlighting a shared global shape landscape rather than distinct macromolecule-specific geometries. (B) Median-based partitioning of the pocket shape space into morphological archetypes. Same shape space as in (A) with pockets colored according to their assigned morphological archetype: sphere-like, rod-like, disk-like, and strongly anisotropic. Archetypes are defined using the global medians of *rEV21* and *rEV32* computed on the combined RNA–protein dataset, resulting in a relative, scale-independent partitioning of the continuous shape space. Each archetype contains both RNA and protein pockets, indicating that dominant pocket morphologies are shared across macromolecular types. (C) Distribution of RNA and protein pockets within each archetype. Same archetypal partitioning as in (B), with RNA and protein pockets shown separately to illustrate their respective contributions to each morphological category. Representative pocket examples are annotated to highlight the structural diversity encompassed within each archetype. This panel emphasizes that, although the relative frequencies may differ, RNA and protein pockets populate the same archetypal regions of shape space.

As illustrated in Fig. 3B, all 4 archetypes are populated by both RNA and protein pockets, indicating that the dominant morphometric regimes are shared across macromolecular types. Importantly, boundaries between archetypes correspond to operational divisions within a continuous shape space rather than sharp geometric discontinuities. Archetypes should therefore be interpreted as representative regions of morphometric space rather than as discrete or mutually exclusive pocket categories. We next characterize the morphometric properties of these 4 archetypes using shape and size descriptors.

#### Morphometric interpretation of the 4 pocket archetypes

Integration of shape- and size-related descriptors by archetype (Table 2) enables a coherent morphometric characterization of the dominant pocket geometries observed in RNA and protein datasets. Archetypes are primarily defined by relative anisotropy patterns captured by eigenvalue ratios, while size-related descriptors provide complementary information on pocket extent (Section S9).

**Table 2.** Morphometric and size-related descriptors of RNA and protein binding pockets across morphological archetypes. For each morphological archetype, descriptive values are reported for the 3 eigenvalue-ratio shape descriptors (*rEV21*, *rEV32*, and *rEV31*), and the 2 size-related descriptors (number of atoms and radius of gyration). Values are provided as means (± SD) for RNA pockets, protein pockets, and the combined RNA + protein dataset, together with the median of the combined distribution. Combined values are reported for descriptive purposes only.

Archetype	Descriptor	RNA mean (SD)	Protein mean (SD)	RNA + Protein mean (SD)	RNA + Protein median
Sphere-like	*rEV21*	0.70 (0.08)	0.72 (0.09)	0.72 (0.09)	0.69
	*rEV32*	0.69 (0.09)	0.72 (0.09)	0.71 (0.09)	0.70
	*rEV31*	0.49 (0.07)	0.52 (0.10)	0.51 (0.09)	0.49
	*Number of atoms*	288.33 (72.69)	202.63 (44.04)	233.73 (69.52)	222.00
	*Radius of gyration*	9.49 (0.93)	8.86 (0.80)	9.09 (0.90)	9.09
	*Number of pockets*	49	86	135	-
Disk-like	*rEV21*	0.73 (0.09)	0.73 (0.10)	0.73 (0.09)	0.71
	*rEV32*	0.41 (0.12)	0.45 (0.09)	0.43 (0.11)	0.45
	*rEV31*	0.30 (0.09)	0.32 (0.08)	0.31 (0.08)	0.31
	*Number of atoms*	234.03 (68.84)	164.66 (51.73)	202.92 (70.63)	197.00
	*Radius of gyration*	9.04 (0.94)	8.48 (1.01)	8.79 (1.01)	8.71
	*Number of pockets*	91	74	165	-
Strongly anisotropic	*rEV21*	0.43 (0.13)	0.46 (0.11)	0.44 (0.12)	0.47
	*rEV32*	0.42 (0.11)	0.41 (0.11)	0.42 (0.11)	0.43
	*rEV31*	0.18 (0.07)	0.19 (0.07)	0.18 (0.07)	0.19
	*Number of atoms*	209.60 (103.97)	150.07 (60.99)	183.14 (92.14)	174.00
	*Radius of gyration*	8.97 (1.59)	8.74 (1.37)	8.87 (1.49)	9.12
	*Number of pockets*	75	60	135	-
Rod-like	*rEV21*	0.46 (0.10)	0.46 (0.09)	0.46 (0.10)	0.49
	*rEV32*	0.73 (0.10)	0.76 (0.11)	0.74 (0.10)	0.75
	*rEV31*	0.34 (0.09)	0.35 (0.09)	0.34 (0.09)	0.34
	*Number of atoms*	251.99 (98.55)	178.64 (60.19)	221.58 (92.01)	213.00
	*Radius of gyration*	9.57 (1.46)	8.93 (1.05)	9.26 (1.32)	9.20
	*Number of pockets*	85	80	165	-

Sphere-like pockets are characterized by high *rEV21* and *rEV32* values (≈ 0.7), indicating near-isotropic geometries with comparable dispersion along the 3 principal inertia axes.

Disk-like pockets combine high *rEV21* with markedly lower *rEV32* values, reflecting flattened geometries resulting from compression along 1 secondary axis.

Rod-like pockets display the opposite pattern, with lower *rEV21* and higher *rEV32* values, corresponding to strong elongation along a single dominant axis while maintaining relative symmetry across the minor axes.

Strongly anisotropic pockets occupy the most extreme region of the morphometric space, with low values of both *rEV21* and *rEV32*, indicating pronounced anisotropy across all axes.

Together, these archetypes define a continuous gradient of anisotropy in the (*rEV21*, *rEV32*) space, ranging from near-isotropic to highly anisotropic geometries. The consistency of these numerical ranges across RNA and protein datasets supports the robustness of the median-based partitioning scheme.

Size-related descriptors reveal additional trends across morphological archetypes. The number of atoms increases progressively from strongly anisotropic to sphere-like pockets, indicating a gradual increase in pocket size across the shape spectrum. In contrast, the radius of gyration exhibits greater variability across archetypes. Rod-like and sphere-like pockets tend to display higher and more dispersed radius of gyration values than disk-like and strongly anisotropic pockets, reflecting a more extended spatial distribution of pocket atoms within these archetypes.

These variations reflect differences in overall pocket extension rather than changes in relative shape anisotropy, which remains conserved within each archetype by construction.

### RNA and protein pocket archetype similarity: Distance and multivariate analysis

#### Pairwise distances between RNA and protein pocket archetypes

Pairwise distances between individual RNA and protein pockets were first analyzed independently of archetype assignment. The averages of pairwise distances computed between all RNA–RNA, protein–protein, and RNA–protein pocket pairs are highly similar (0.356 ± 0.179, 0.358 ± 0.184, and 0.362 ± 0.186). Not only central tendencies but also distribution tails were comparable. In particular, the lower 5% quantiles were nearly identical for RNA–RNA, protein–protein, and RNA–protein most similar pocket pairs: 0.094, 0.092, and 0.094. These results confirm that RNA and protein pockets are embedded within a common morphometric space rather than forming segregated shape populations and that highly similar pocket shape pairs occur both within and across macromolecular types.

**Fig. 4.**
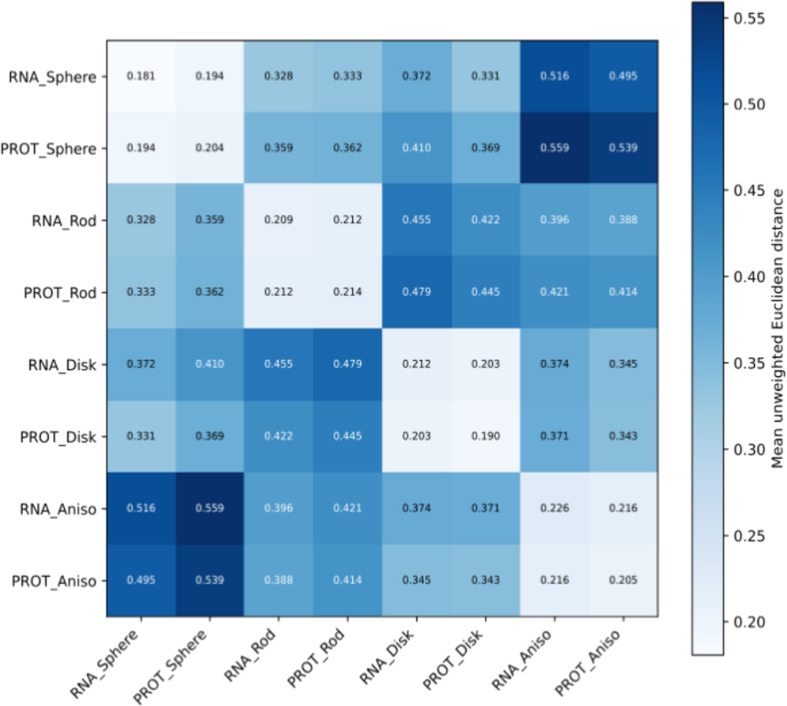
Intra- and interarchetype morphometric distances between RNA and protein binding pockets. Distances along the diagonal correspond to within-archetype morphometric dispersion for RNA and protein pockets separately, whereas off-diagonal values represent interarchetype morphometric distances.

To assess the geometric correspondence between RNA and protein archetypes, we computed mean unweighted Euclidean distances between all pairs of pockets within and between the 4 morphological archetypes according to their associated macromolecules. An 8×8 heatmap of mean unweighted Euclidean distances (Fig. 4) summarizes these results, highlighting strong intra-archetype cohesion, minimal distances between corresponding RNA and protein archetypes, and conserved distance hierarchies across macromolecular types.

Diagonal values of the distance matrix correspond to within-archetype, within-macromolecule distances (RNA–RNA and protein–protein separately) and provide a measure of archetype compactness. These intra-archetype distances are consistently low, ranging from approximately 0.18 to 0.23 for RNA pockets and from 0.19 to 0.21 for protein pockets, indicating comparable levels of internal morphometric cohesion across macromolecular types. Among archetypes, strongly anisotropic pockets exhibit slightly higher internal dispersion, consistent with their broader spread in morphometric space.

Distances between RNA and protein pockets assigned to the same archetype are of similar magnitude (≈ 0.19 to 0.22), indicating a strong geometric correspondence between RNA and protein pockets within corresponding morphometric regimes. These cross-macromolecular, within-archetype distances are comparable to the intrinsic variability observed within RNA-only or protein-only pockets.

In contrast, distances between pockets belonging to different archetypes are substantially larger, with mean values around 0.40 and reaching up to ~0.55. This 2-fold increase relative to within-archetype distances demonstrates that the 4 archetypes correspond to quantitatively distinct regions of the continuous shape space, despite the absence of sharp geometric boundaries. The distance matrix further reveals a conserved hierarchy in which sphere-like and strongly anisotropic archetypes define the largest separations across both RNA and protein datasets (≈ 0.52 to 0.56), whereas intermediate archetypes such as rod-like and disk-like pockets display more moderate separations.

Together, these distance-based analyses show that the median-defined archetypes capture conserved geometric regimes shared by RNA and protein binding pockets, while maintaining sufficient separation to support comparative analyses across shape classes.

Because morphometric distances are descriptive in nature, we next used multivariate statistical analyses to formally test whether macromolecular identity introduces additional shape variation within corresponding archetypes.

#### Absence of systematic RNA–protein shape differences within archetypes

Multivariate differences between RNA and protein pocket shapes within each morphological archetype were assessed using MANOVA on the 2 independent eigenvalue ratios *rEV21* and *rEV32*. Across all 4 archetypes, no significant RNA–protein differences were detected (Pillai’s trace; Section S9.1), indicating that relative pocket anisotropy is conserved between macromolecular types once global shape is accounted for. Complementary univariate analyses confirmed the absence of systematic RNA–protein differences for *rEV21* within archetypes. A marginal trend was observed for disk-like pockets, associated with a modest univariate difference in *rEV32* (Cohen’s *d* ≈ −0.37), but this localized effect did not translate into a significant multivariate shape divergence.

In contrast, size-related descriptors exhibited consistent and significant RNA–protein differences across archetypes. For all 4 morphometric archetypes, RNA pockets contain a larger number of atoms than their protein counterparts, with large effect sizes (Cohen’s *d* generally > 0.7). Beyond atomic counts, the radius of gyration reveals a more nuanced, archetype-dependent behavior. For sphere-like, disk-like, and rod-like archetypes, RNA pockets tend to display a more extended spatial distribution of pocket atoms, with mean radius of gyration values approximately 0.5 to 0.7 Å larger than those of protein pockets, corresponding to small to moderate effect sizes. By contrast, strongly anisotropic pockets show largely overlapping radius of gyration distributions between RNA and protein pockets, with negligible effect sizes. Importantly, although RNA and protein pockets differ in size, their relative shape anisotropy remains comparable within each archetype, indicating that variations in radius of gyration capture pocket extent rather than shape. Detailed statistical results are provided in Section S9.2.

Having shown that RNA and protein pockets assigned to the same archetype are morphometrically comparable, we next asked whether global RNA–protein differences instead arise from differential occupations of these shared shape archetypes.

### Distribution of pocket shape archetypes in RNA and protein datasets

While multivariate analyses revealed only subtle RNA–protein differences in continuous shape descriptors within each archetype, we next examined whether global RNA–protein differences instead arise from differences in the relative frequencies of morphological archetypes rather than from systematic geometric shifts within archetypes.

**Fig. 5.**
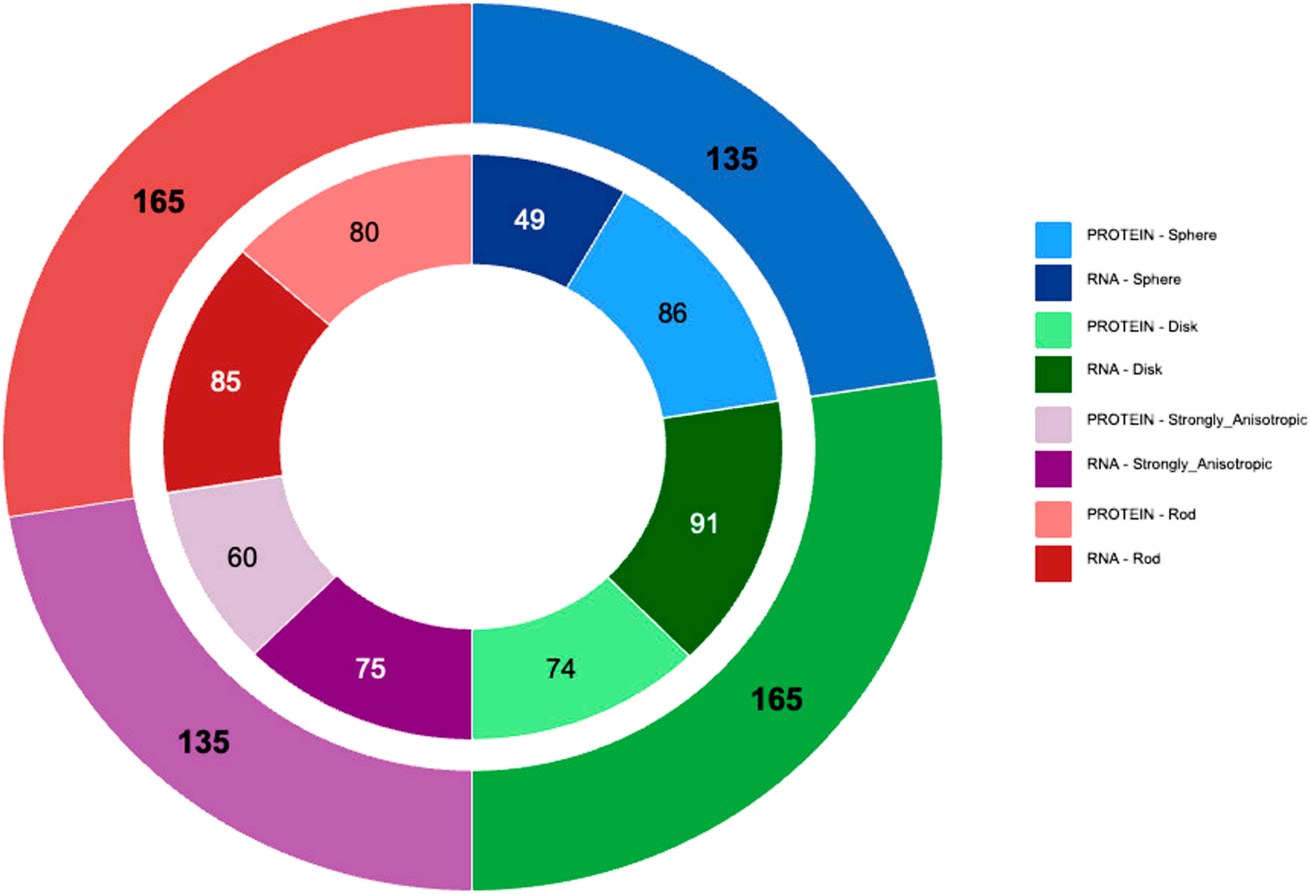
Distribution of RNA and protein pockets across morphological archetypes. Donut representation of pocket shape archetype frequencies in RNA and protein datasets. The outer ring shows the global distribution of the 4 morphological archetypes in the combined dataset, with numbers indicating total counts per class. The inner ring displays the relative contributions of RNA and protein pockets within each archetype, with each sector split according to macromolecular type. The outer ring indicates the corresponding absolute pocket counts for each archetype. Sphere-like pockets are proportionally more frequent in proteins, whereas disk-like and strongly anisotropic pockets are relatively enriched in RNA. Rod-like pockets exhibit comparable frequencies in both macromolecular types, supporting the existence of shared morphological regimes within a common morphometric space.

Application of the median-based decision tree shows that all 4 morphological archetypes are substantially populated in both RNA and protein datasets, confirming the existence of a shared morphometric shape space across macromolecular types (Fig. 5). In the combined dataset, rod-like and disk-like pockets are the most frequent morphologies (each 27.5%), whereas sphere-like and strongly anisotropic pockets each account for 22.5% of pockets (Table 3).

**Table 3.** Distribution of morphological pocket archetypes in RNA and protein datasets. Counts and percentages of pockets assigned to each morphological archetype in RNA and protein datasets (*n* = 300 each), as well as in the combined dataset (*n* = 600). Percentages are reported relative to each macromolecular dataset and to the total population.

Archetype	RNA	Protein	Global
Sphere-like	49 (16.3%)	86 (28.7%)	135 (22.5%)
Disk-like	91 (30.3%)	74 (24.7%)	165 (27.5%)
Strongly anisotropic	75 (25.0%)	60 (20.0%)	135 (22.5%)
Rod-like	85 (28.3%)	80 (26.7%)	165 (27.5%)
Total	300	300	600

When RNA and protein pockets are considered separately, clear differences in archetype frequencies emerge (χ^2^ test, *P* ≪ 0.01; Table 3). Sphere-like pockets constitute the most frequent class in proteins but represent the least frequent archetype in RNA, whereas disk-like pockets and strongly anisotropic pockets are preferentially enriched in RNA. In contrast, rod-like pockets exhibit nearly identical frequencies in both macromolecular datasets, indicating a conserved contribution of this archetype across RNA and protein binding sites.

Importantly, these differences reflect population-level enrichment rather than archetype-specific shape divergence, as RNA and protein pockets assigned to the same archetype remain morphometrically similar. Global RNA–protein differences in eigenvalue-ratio distributions therefore primarily arise from differential occupations of shared morphometric regimes rather than from systematic geometric reorganization within archetypes.

These results further support the interpretation that RNA and protein pockets share the same morphometric space but differ in their relative usage of these shape archetypes. Archetype frequencies are summarized numerically in Table 3 and visually in Fig. 5.

### Ligand physicochemical properties across pocket shape archetypes

To assess whether morphometric archetypes are associated with specific ligand chemical properties, we analyzed ligand physicochemical descriptors (molecular weight, QED, ALOGP, polar surface area, and aromatic ring count) across morphometric pocket shape archetypes (Section S10). While a statistically significant multivariate signal was detected within both RNA and protein datasets, descriptor distributions showed substantial overlap across archetypes, and effect sizes remained small to moderate. No archetype exhibited a distinct physicochemical profile or exclusive chemotype enrichment. These results indicate that morphometric classes are not trivially defined by ligand chemistry and do not correspond to chemically segregated ligand families within the present dataset.

### Structural interpretation of morphological pocket archetypes

#### Illustration of representative RNA and protein pockets across morphological archetypes

To provide an intuitive structural interpretation of the morphometric archetypes identified in the *rEV21*–*rEV32* shape space, we selected 1 representative RNA and 1 representative protein binding pocket for each of the 4 archetypes. These examples are intended solely to illustrate how numerically defined regions of the shape space can correspond to distinct 3-dimensional pocket geometries, rather than to establish general structural rules for RNA or protein binding sites.

Representative pockets were chosen among extreme but unambiguous members of each archetype, located away from the median-based decision boundaries in the *rEV21*–*rEV32* morphospace. This selection strategy ensures that the illustrated cases correspond to well-defined regions of the continuous morphometric landscape described in Section 3.2, while avoiding ambiguous or borderline assignments. Their positions in the numerical shape space are shown in Fig. 3C, illustrating that RNA and protein pockets can be selected symmetrically within each archetype and occupy overlapping regions of the same morphometric framework.

#### Structural interpretation by archetype

**Fig. 6.**
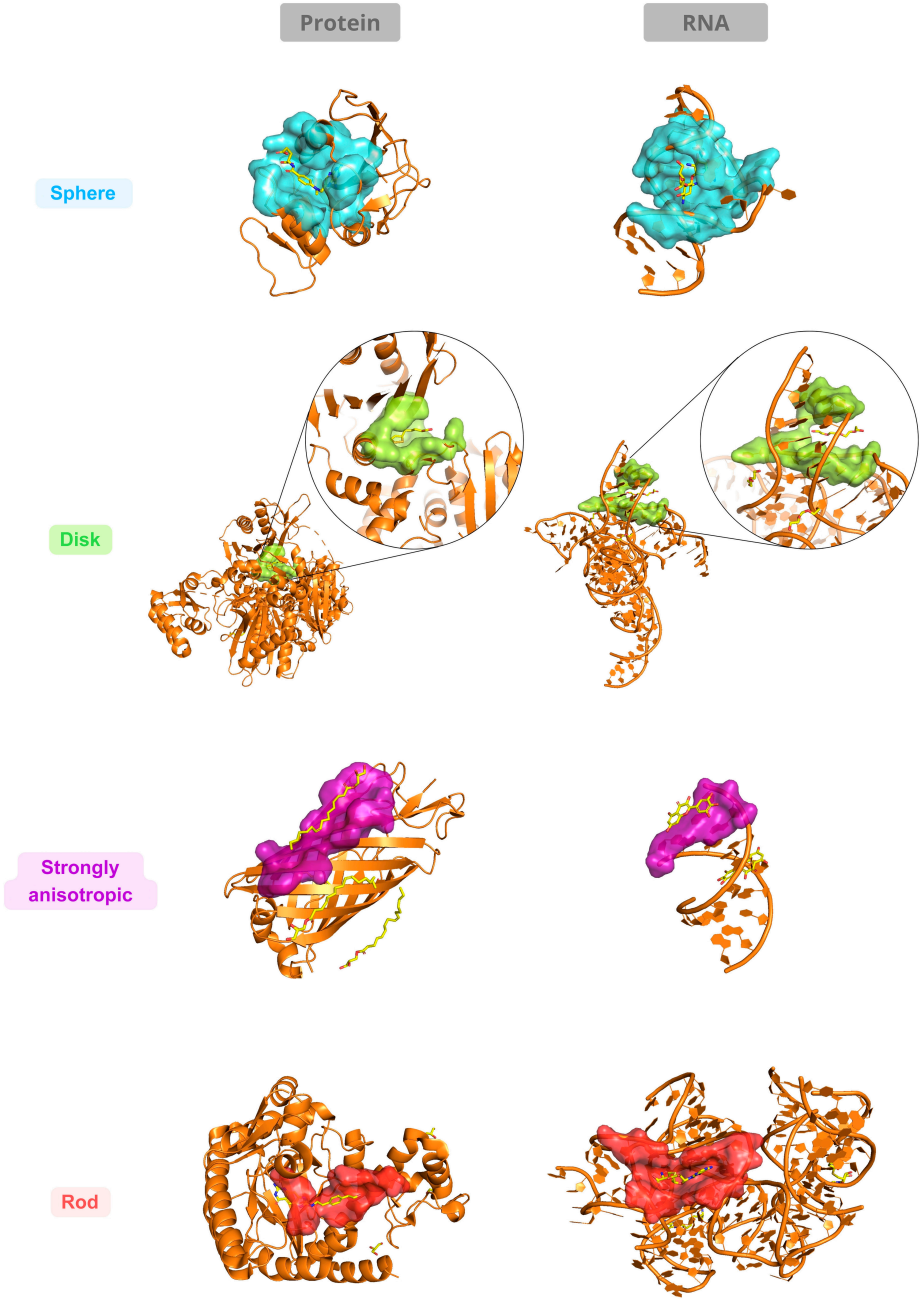
Structural illustrations of representative RNA and protein binding pockets across morphological archetypes. For each of the 4 morphological archetypes (sphere-like, disk-like, strongly anisotropic, and rod-like), 1 representative protein binding pocket and 1 representative RNA binding pocket are shown. Representative examples correspond to the following PDB–ligand complexes: sphere-like (protein: 2qk8_MTX; RNA: 2n0j_RIO), disk-like (protein: 1x2h_LPA; RNA: 8f4o_PG4), strongly anisotropic (protein: 5g26_97N; RNA: 5xi1_MYC), and rod-like (protein: 5jxq_6OK; RNA: 3npq_SAH). Pockets were selected as unambiguous examples located away from the median-based decision boundaries in the *rEV21*–*rEV32* morphometric space (Fig. **3**C). Pocket surfaces are displayed together with their corresponding macromolecular backbones and bound ligands. These structural illustrations provide qualitative structural context for the defined archetypes and highlight that RNA and protein pockets occupy overlapping regions of the same shape space, without implying exhaustiveness or archetype-defining structural features.

Figure 6 presents 3-dimensional visualizations of the selected RNA and protein pockets for each archetype, displayed side by side to facilitate qualitative comparison. In the following sections, archetypes are discussed one by one, with emphasis on geometric organization and local structural context, without implying exhaustiveness or exclusivity of the illustrated features.

##### Sphere-like archetype

In the RNA example shown in Fig. 6, the pocket displays a compact 3-dimensional shape consistent with near-isotropic eigenvalue ratios. Pocket-forming atoms originate from multiple structural elements, including stems and loop regions, reflecting a locally convergent organization of RNA backbone segments. This observation illustrates how quasi-isotropic pocket shapes can arise in RNA structures without being restricted to a single secondary-structure motif. The protein example exhibits a similarly compact shape, with pocket atoms distributed relatively evenly along the 3 principal axes. In this case, the near-isotropic shape results from the local packing of secondary-structure elements, producing a cavity whose shape is not dominated by a single preferred direction.

Taken together, these illustrative cases show that the sphere-like archetype corresponds to a morphometric signature of near-isotropic pocket shapes, characterized by balanced spatial dispersion along the 3 principal axes and observed in both RNA and protein structures, independently of macromolecular type or specific structural context.

##### Disk-like archetype

In the RNA example shown in Fig. 6, the disk-like pocket displays a flattened overall shape together with a locally heterogeneous cross-sectional organization. This visual appearance is consistent with the reduced *rEV32* values observed for this archetype and illustrates how compression along the minor axis can coexist with local shape irregularities in RNA pockets. The protein example exhibits a similarly flattened shape, with a more regular distribution of pocket atoms within the compressed dimension. Despite these local differences in cross-sectional organization, both RNA and protein examples occupy the same region of the morphometric space defined for disk-like pockets.

In conclusion, this shows that disk-like archetype corresponds to a shared morphometric signature defined by anisotropic flattening along 1 axis, while allowing for local variations in shape organization that do not alter the global archetype assignment.

##### Strongly anisotropic archetype

Instead, anisotropy is simultaneously expressed along all 3 principal directions. In the RNA example, the pocket arises from paired helical regions, where base pairing imposes strong directional constraints on the local geometry. Pocket-forming atoms are contributed primarily by stem or stem-end motifs, resulting in a narrow and highly elongated cavity aligned with the helical axis. The protein example displays a similarly anisotropic geometry in a distinct macromolecular context, with the pocket forming along the surface of a α-helical bundle. In both illustrative cases, the pockets are located at the molecular surface, where geometric constraints arise mainly from local backbone organization rather than from enclosure within a compact cavity. These case studies illustrate how distinct structural arrangements can converge toward similar strongly anisotropic morphometric signatures.

Importantly, these cases highlight the fact that strongly anisotropic archetype corresponds to a consistent set of geometric properties, characterized by minimal symmetry and simultaneous anisotropy along all 3 principal axes, independent of macromolecular type, chemical composition, or specific binding mechanisms.

##### Rod-like archetype

In the representative RNA and protein examples shown in Fig. 6, rod-like pockets display pronounced axial elongation aligned with locally elongated structural features. In this case, the rod-like shape arises from the local arrangement of secondary-structure elements, producing a groove- or channel-like cavity whose shape is not dominated by compression along the secondary axes. Overall, these illustrative cases show that the rod-like archetype corresponds to a morphometric signature defined by strong axial elongation combined with relative symmetry across the minor axes, observed in both RNA and protein pockets.

Together, these illustrative examples demonstrate that similar pocket shapes, as defined by morphometric descriptors, can arise from diverse structural contexts in both RNA and protein systems. Importantly, they are presented as concrete visual support for the quantitative classification rather than as exhaustive or statistically representative structural assignments.

They confirm that RNA and protein binding pockets populate the same dominant morphological archetypes. Within each archetype, pocket shape is largely conserved across macromolecular types, while RNA–protein differences primarily involve pocket size and local shape regularity rather than changes in global morphometric class.

While archetype-based classification provides a structured and interpretable partitioning of the shape space, the underlying morphometric framework remains continuous. Accordingly, continuous distance measures offer a complimentary means to compare individual pockets, particularly for cases located near archetype boundaries.

### Limitations and interpretation of boundary cases

Although pocket morphologies were discretized into 4 archetypal classes using global median thresholds, the underlying eigenvalue-ratio space is continuous. As a result, pockets located near decision boundaries may be assigned to different morphological archetypes despite exhibiting highly similar geometric profiles.

**Fig. 7.**
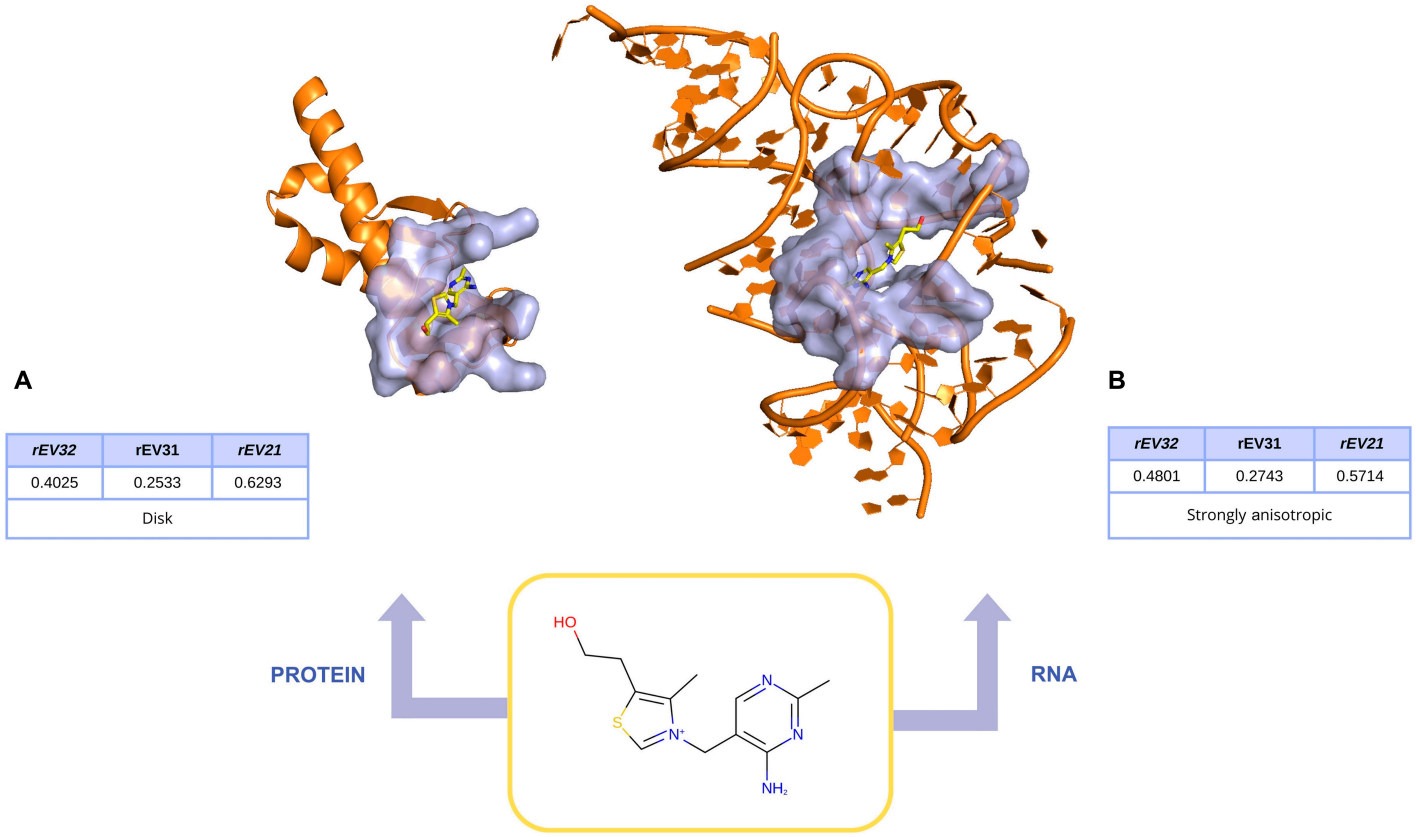
Boundary case illustrating morphometric similarity despite different archetype assignments. Representative protein and RNA binding pockets extracted from complexes involving the same ligand, thiamin (vitamin B1, VIB), are shown (protein: PDB 2lh8, [A]; RNA: PDB 4nyg, [B]). Based on their relative positions in the *rEV21*–*rEV32* shape space, the protein pocket is assigned to the strongly anisotropic archetype, whereas the RNA pocket is assigned to the disk-like archetype. Despite this difference in archetype assignment, both pockets exhibit highly similar eigenvalue ratios, resulting in a small unweighted Euclidean distance in the 3-dimensional ratio space (*d* ≈ 0.099).

To illustrate this effect, we examined thiamin (vitamin B1, VIB), a ligand known to bind both RNA and protein targets. Thiamin binds to a disk-like pocket in ribosomal RNA and to a protein pocket classified as strongly anisotropic. Their relative positions in the morphometric shape space are shown in Fig. 3C, and the corresponding structures are illustrated in Fig. 7. Despite their different macromolecular contexts and archetype assignments, both pockets display highly similar eigenvalue ratios, resulting in a small unweighted Euclidean distance in the 3-dimensional ratio space (*d* ≈ 0.099), close to the lower tail of the RNA–protein pocket distance distribution (5th percentile ≈ 0.094).

This boundary case illustrates that morphometrically similar pockets may receive different archetype assignments when they lie close to partition thresholds, whereas continuous distance-based descriptors more faithfully capture their underlying geometric similarity. This example is intended solely as a methodological illustration of the continuity of the pocket shape space and of the complementarity between discrete archetype partitioning and continuous morphometric descriptors, rather than as a statement on ligand promiscuity.

Although the present framework deliberately focuses on pocket geometry independently of specific ligands, molecular recognition ultimately arises from the interplay between pocket shape and ligand chemical properties. In this context, inertia-based descriptors should be viewed as a geometric reference for comparative analyses rather than as standalone predictors of binding behavior.

## Discussion

We aimed to provide a unified and quantitative framework to compare RNA and protein binding pockets based on their 3-dimensional shape anisotropy. While protein binding sites have been extensively characterized using a wide range of geometric and physicochemical descriptors [18–20], the geometric organization of RNA binding pockets remains far less systematically explored.

Previous shape-based approaches relied on simplified descriptions of pocket geometry, typically defined by 2 principal axis ratios (*λ*_2_/*λ*_1_ and *λ*_3_/*λ*_1_) derived from the eigenvalues of the inertia matrix and projected onto a 2-dimensional shape space. Such frameworks, initially applied to protein binding sites [1,2] and later extended to RNA pockets [4], enabled the classification of molecular pockets into 3 main categories: sphere-like, rod-like, and disk-like.

However, this biplanar representation omits the local anisotropy ratio *λ*_3_/*λ*_2_, which captures symmetry within the minor plane and is essential to distinguish axially elongated rod-like shapes from flattened, strongly anisotropic geometries. Incorporating this third ratio enables a more complete decomposition of pocket anisotropy and leads to a 4-regime organization of the morphometric space, encompassing sphere-like, disk-like, rod-like, and strongly anisotropic pocket shapes. Building on this extension, we introduce a size-independent morphometric framework designed to compare ligand-binding pocket shapes across RNA and protein structures within a common geometric space. By relying exclusively on eigenvalue ratios derived from the inertia matrix, the framework captures relative anisotropy independently of absolute pocket size or macromolecular composition. This enables direct quantitative comparisons across distinct macromolecular contexts without embedding protein- or RNA-specific assumptions.

Here, we clarify the scope, assumptions, and limitations of inertia-based descriptors used throughout this study. Although inertia-based descriptors provide a compact and algebraically grounded summary of global pocket geometry, their scope and limitations must be explicitly acknowledged. By construction, eigenvalue ratios capture only second-order spatial moments of the pocket point cloud and therefore describe global anisotropy rather than fine-grained local features such as surface roughness, pocket branching, or interaction-specific motifs. Consequently, these descriptors summarize the overall spatial organization of binding sites and should be interpreted as global geometric descriptors rather than exhaustive structural characterizations.

Inertia-based descriptors are known to be sensitive to isolated or extreme coordinates. In the present framework, this sensitivity is reduced by the ligand-centered definition of binding pockets. In this definition, pocket atoms are selected based on their spatial proximity to a bound ligand. These atoms then form a contiguous and localized region of the macromolecular structure. This effect is further reinforced by the residue-level pocket definition, which preserves structural continuity and limits fragmentation, particularly in RNA binding sites where ligand recognition often involves spatially separated nucleotides brought together by tertiary folding.

The inertia matrix was deliberately computed using pocket heavy atoms only and without atom-type weighting in order to focus exclusively on geometric organization. Because all pocket comparative analyses were performed under homogeneous hydrogen-free conditions, this methodological choice does not introduce bias in the RNA–protein comparison and ensures structural consistency across macromolecular types. A sensitivity analysis further confirmed that eigenvalue ratios are only marginally affected by hydrogen inclusion when pockets involve the same set of residues.

The framework is strictly geometric and descriptive: No functional, physicochemical, or ligand-chemistry information is used to define the shape descriptors or construct the morphometric space. It should therefore be regarded as a geometric reference for reducing structural heterogeneity and comparing pocket shapes, rather than as a predictor of affinity, specificity, or druggability.

Because pocket estimation is ligand-centered and based on experimentally resolved bound conformations, the morphometric landscape described here reflects stabilized binding site geometries rather than the full spectrum of conformationally accessible cavities. As a result, the framework captures bound-state geometry rather than intrinsic pocket plasticity, which would require systematic apo–holo comparisons or explicit conformational sampling. Morphometric archetypes are therefore not intended to infer binding mechanisms or functional states but to provide a structural reference upon which future mechanistic or dynamical investigations may build. The comparative analysis therefore reveals robust geometric patterns shared across RNA and protein binding pockets.

Importantly, pocket shapes are not interpreted relative to idealized geometric objects such as perfect spheres or cylinders. Instead, they are positioned within the empirical distribution of geometries observed in biological binding sites. Within this empirical context, intermediate anisotropy values correspond to concave, open, and solvent-exposed cavities, a defining feature of ligand-binding pockets that is poorly captured by comparisons to closed or idealized shapes. Inertia-based descriptors thus provide an abstract yet biologically meaningful representation of pocket geometry that accommodates both concavity and openness.

Within this purely geometric and size-independent framework, a key outcome of the analysis is the clear decoupling between pocket shape and pocket size. While eigenvalue ratios reveal conserved relative shape organization between RNA and protein pockets within each morphometric regime, RNA pockets are consistently larger in absolute terms, as reflected by higher atom counts and, to a lesser extent, larger radii of gyration. In this context, the radius of gyration provides a complementary measure of pocket extent by quantifying the average spatial dispersion of pocket atoms around their center of mass. Differences in radius of gyration therefore reflect variations in overall spatial reach rather than changes in pocket shape. Together, these observations indicate that pocket shape and size encode complementary but distinct dimensions of binding pocket architecture.

Beyond discrete archetype-based descriptions, it is important to emphasize that the underlying morphometric space is continuous. In this discussion, geometric regimes refer to continuous regions of this space, whereas archetypes denote their operational discretization based on global median-derived thresholds. Because the morphometric space defined by eigenvalue ratios is continuous and bounded, the present framework uses a deterministic median-based partitioning that provides stable and interpretable reference regions, rather than relying on parameter-dependent unsupervised clustering approaches whose solutions may vary with sampling and preprocessing. Robustness analyses further indicate that this partitioning reflects stable regions of the underlying shape space shared by RNA and protein pockets, rather than algorithm-specific cluster assignments. This highlights the complementarity between discrete archetype-based descriptions and continuous distance-based measures: While archetypes offer an interpretable organization of the shape space, distance metrics remain essential for capturing fine-grained geometric similarity, particularly near regime boundaries. Pockets located near regime boundaries may exhibit highly similar eigenvalue-ratio profiles despite belonging to different archetypes, underscoring the value of continuous morphometric distances alongside categorical labels.

Together, these observations further support the view that RNA and protein binding pockets occupy a shared continuous morphometric space organized into common geometric regimes.

Within this framework, we examined whether RNA and protein binding pockets occupy distinct regions of the morphometric space or share common geometric regimes. The results indicate that RNA and protein pockets largely populate the same regimes. Multivariate analyses performed within each regime reveal no systematic RNA–protein divergence in eigenvalue-ratio descriptors once global geometry is controlled for. In particular, global elongation and cross-sectional symmetry are conserved between RNA and protein pockets within corresponding regimes.

Importantly, this similarity is not imposed by the partitioning scheme itself: The median-based thresholds do not enforce equivalence between RNA and protein pockets, and any intrinsic divergence would manifest as systematic shifts in eigenvalue-ratio distributions or increased morphometric distances within regimes. The absence of such effects supports the conclusion that RNA and protein pockets genuinely share dominant geometric organizations.

While no systematic RNA–protein shape differences are detected within morphometric regimes in the present dataset, subtle effects could emerge as larger and more diverse datasets become available. If present, such effects would likely reflect fine-grained variations within a largely shared geometric framework rather than fundamental shape divergence.

While these morphometric regimes are shared, RNA and protein pockets differ in their relative frequencies across them. Frequency analyses indicate that sphere-like pockets are more prevalent in proteins, whereas disk-like and strongly anisotropic pockets are enriched in RNA, with rod-like pockets occurring at comparable frequencies in both datasets. These global RNA–protein differences therefore arise from differential occupancy of shared morphometric regimes, rather than from intrinsic geometric divergence within regimes. To control for potential biases related to RNA structural composition, we further examined the distribution of morphometric archetypes across major RNA structural classes, including regulatory RNAs, riboswitches, structured functional RNAs, mRNAs, and ribosomal RNAs. No single RNA class was found to dominate any archetype, indicating that the observed shape distributions are not driven by overrepresentation of specific RNA families (Section S11). These frequency patterns should be interpreted in the context of the present dataset, but they consistently point to distinct shape usage preferences between RNA and protein binding pockets.

For protein binding sites, this distribution is consistent with previous observations that ligandable protein cavities tend to be relatively compact and well defined. Earlier shape-based analyses did not explicitly define discrete morphometric archetypes, but rod-like and disk-like geometries appear broadly balanced in protein datasets, with near-isotropic pockets remaining frequent, particularly among druggable sites [21].

Importantly, pockets classified here as strongly anisotropic do not correspond to marginal or ill-defined cases in morphometric space. Strongly anisotropic pockets represent a substantial fraction of both RNA and protein datasets. In the principal component analysis representation (Fig. 3), the strongly anisotropic pockets occupy a distinct and well-defined region of the morphometric space and do not appear more dispersed than other archetypes. This indicates that the strongly anisotropic regime forms a stable component of the shared RNA–protein morphometric space rather than a marginal or boundary artifact. Their systematic presence in both RNA and protein datasets further supports the relevance of this regime as a meaningful component of the morphometric landscape.

Consistently, this regime is observed across both RNA and protein pockets and is not driven by a single RNA structural class. As shown in Section S11, strongly anisotropic RNA pockets are enriched in riboswitches and structured functional RNAs, while ribosomal RNAs do not dominate this category. This distribution supports the interpretation that strong anisotropy reflects generic geometric constraints associated with narrow and spatially constrained binding environments, rather than artifacts arising from a specific macromolecular class or dataset bias.

To facilitate interpretation of the morphometric space, we examined representative structural examples for each regime. These illustrations are intended to provide geometric intuition rather than to imply systematic functional or mechanistic assignments. Disk-like pockets correspond to flattened geometries compressed along 1 principal axis, whereas rod-like pockets display pronounced axial elongation combined with relative symmetry across the minor axes. Sphere-like pockets correspond to near-isotropic geometries characterized by balanced spatial dispersion. Strongly anisotropic pockets lack a dominant symmetry axis and are observed in both RNA and protein systems. They can occur at peripheral or geometrically constrained regions of the macromolecular structure, as illustrated in Fig. 6.

Together, these examples illustrate how distinct structural and architectural contexts can converge toward similar global geometric signatures. This convergence illustrates how inertia-based shape descriptors capture overarching patterns of pocket geometry, regardless of local structural details or macromolecular composition.

Several recent studies have explored RNA binding-pocket characterization using complementary methodological perspectives. The RPocket database provides a large-scale structural inventory of RNA ligand-binding sites based on geometric descriptors derived from principal axis ratios, enabling classification into sphere-, rod-, and disk-like categories [4]. The present framework extends this representation by incorporating an additional anisotropy ratio, allowing resolution of strongly anisotropic geometries and enabling direct comparison of RNA and protein binding pockets within a unified morphometric space.

Recent work using the fpocketR framework [22] further examined RNA pocket geometries using normalized principal moments of inertia to classify pockets into rod-, disc-, and sphere-like categories. RNA pockets were reported to predominantly occupy flattened regions spanning rod-like to disc-like geometries. Importantly, this analysis focuses on pockets preselected for favorable ligand-binding properties, thereby characterizing a ligandability-enriched subset of RNA binding environments. Within this context, our observations are geometrically consistent with these findings while extending resolution by separating flattened geometries into disk-like and strongly anisotropic regimes, which remain merged within 3-class normalized principal moments of inertia representations.

Approaches based on Statistical Molecular Interaction Fields [21] further provide a complementary perspective by explicitly comparing RNA and protein binding environments through interaction-energy landscapes and physicochemical descriptors. While such methods capture energetic determinants of molecular recognition across macromolecular systems, they operate at a different descriptive level than the geometry-centered framework proposed here. The present approach instead focuses on size-independent geometric organization, providing a morphometric reference space independent of interaction energetics that may complement interaction- or energy-based comparative analyses.

Taken together, these approaches highlight the emergence of complementary strategies for RNA binding site characterization, in which geometry-based morphometric organization can serve as an upstream structural framework that may be integrated with interaction- or ligandability-oriented analyses without introducing functional assumptions at the geometric characterization stage.

By establishing a unified and size-independent morphometric description shared across RNA and protein binding pockets, this framework provides a geometric reference for comparative analysis of macromolecular binding site architectures.

## Conclusion, Perspectives, and Limitations

Together, these results establish a size-independent, geometry-based morphometric framework for comparing RNA and protein binding pocket shapes. By demonstrating that RNA and protein pockets share dominant morphometric archetypes and that global differences primarily result from differential archetype occupancy rather than intrinsic shape divergence, this work provides a quantitative basis for cross-macromolecule comparisons of binding site architecture.

More broadly, the framework contributes to reducing structural heterogeneity by organizing diverse binding pockets into a limited number of interpretable geometric regimes, thereby simplifying the structural landscape in which RNA and protein pocket diversity can be systematically characterized. However, morphology alone cannot capture the full diversity of ligand-binding environments. Pockets with similar shape profiles may differ substantially in absolute size, electrostatics, polarity, or interaction patterns, all of which critically influence molecular recognition [23–25]. In this respect, inertia-based descriptors should be viewed as a first-level geometric description of pocket organization rather than a description of detailed interaction chemistry and not as a standalone predictor of binding behavior. This interpretation is consistent with previous shape-based analyses of protein binding sites, which emphasized geometry as an organizing but nonsufficient determinant of binding specificity [26].

While the present framework focuses on the geometric organization of binding pockets independently of specific ligands, molecular recognition emerges from the interplay between pocket geometry and ligand properties. The functional relevance of pocket geometry depends on its compatibility with ligand chemical space, suggesting that joint analyses of pocket shape and ligand diversity represent a natural extension of the present framework. By stratifying pockets into continuous morphometric regimes, the framework provides a simplified structural basis upon which ligand accommodation patterns and ligandability hypotheses can be examined in a more coherent manner. Within this perspective, global pocket anisotropy provides complementary geometric information describing the spatial organization of binding environments. More isotropic geometries approximate enclosed cavities frequently observed in protein binding sites, whereas highly anisotropic pockets correspond to elongated or groove-like environments that impose directional constraints on ligand accommodation, particularly in RNA systems. These geometric features do not determine druggability per se but shape the spatial accommodation landscape available to ligands.

More generally, large-scale analyses of RNA binding pockets remain comparatively recent and less mature than analogous studies on protein binding sites. While the present framework establishes a transferable geometric reference for systematic RNA pocket shape characterization, further studies will be required to refine this description and to relate geometric organization to complementary structural, physicochemical, and functional descriptors. Integrating morphometric information with complementary structural and physicochemical descriptors may provide a more comprehensive view of RNA binding environments and support future investigations of RNA pocket accessibility and ligandability [27,28].

Beyond static geometry, recent reviews further highlight that binding site flexibility, transient pocket formation, and conformational heterogeneity play a central role in ligand recognition and cannot be fully captured by purely static descriptors [29,30]. Future developments should therefore integrate the present shape-based framework with complementary descriptors capturing physicochemical properties and conformational dynamics. Coupling morphometric analysis with molecular dynamics simulations represents a promising direction for extending this framework, as pocket flexibility, transient cavity formation, and conformational selection are known to play a critical role in protein–ligand recognition [31–33]. Extending such dynamic pocket analyses to RNA systems would provide a crucial bridge between static geometry, pocket plasticity, and functional accessibility, enabling a more comprehensive, multiscale characterization of binding pockets across RNA and protein targets.

## Data Availability

The code used to compute eigenvalue-based geometric descriptors of protein pockets is publicly available at: https://github.com/IsPP-Team/pocket-eigenvalue-ratio under an MIT license. The repository includes a Python script for extracting atomic coordinates from PDB pocket files, computing covariance matrix eigenvalues and their ratios (*rEV21*, *rEV31*, and *rEV32*), as well as pocket size descriptors (number of atoms and radius of gyration). Example input data are also provided. Usage instructions are included in the documentation to facilitate reproduction of the analyses reported in this study.

## Supplementary Materials

Supplementary 1 Section S1: Table S1 Section S2: Fig. S2 Section S3: Fig. S3 Section S4: Tables S4.2 and S4.3 Section S5: Table S5 Section S6: Tables S6.1 and S6.2 and Fig. S6 Section S7: Fig. S7 and Table S7.2 Section S8: Fig. S8 Section S9: Tables S9.1, S9.2, and S9.3 Section S10: Fig. S10 and Table S10 Data S11: Table S11
