# Supplementary material for: Eigenvalue Ratios Reveal Shared Binding Pocket Shapes in RNA and Protein Structures
Source: Comput Struct Biotechnol J. 2026 Apr 20;35(1):0022. doi: 10.34133/csbj.0022 (PMC13094404; doi:10.34133/csbj.0022)
Supplement: Supplementary 1 — Section S1: Table S1 Section S2: Fig. S2 Section S3: Fig. S3 Section S4: Tables S4.2 and S4.3 Section S5: Table S5 Section S6: Tables S6.1 and S6.2 and Fig. S6 Section S7: Fig. S7 and Table S7.2 Section S8: Fig. S8 Section S9: Tables S9.1, S9.2, and S9.3 Section S10: Fig. S10 and Table S10 Data S11: Table S11 [file csbj.0022.f1.zip › Supplementary_Data_CSBJ-D-25-01603.pdf]

Supplementary Data

Eigenvalue ratios reveal shared binding pocket shapes in RNA and protein structures

Leïla Ziani[1], Anne Badel[1], Léa Dufay[1], Delphine Flatters[1], Leslie Regad[1], Anne-Claude Camproux[1,2]

Supplementary Section S1 – Table S1 – List of protein complexes included

Supplementary Section S2 – Pocket representation and geometric coherence

Supplementary Section S3 – Ligand chemical space controls

Supplementary Section S4 – Redundancy and dataset-dependence controls

Supplementary Section S5 – Table S5 – Definition of morphometric descriptors

Supplementary Section S6 – Robustness of pocket estimation

Supplementary Section S7 – Sensitivity of inertia-based pocket descriptors to hydrogen inclusion

Supplementary Section S8 – Conserved pocket shape but divergent pocket size across archetypes

Supplementary Section S9 – Multivariate and univariate statistical analyses within morphological archetypes

Supplementary Section S10 – Association between Morphometric Archetypes and Ligand Physicochemical Properties

Supplementary Section S11 – RNA structural class distribution across morphometric archetypes

[1] Université Paris Cité, CNRS UMR 8251 INSERM ERL U1133, Unité de Biologie Fonctionnelle et Adaptative, BFA, F-75013 Paris, France

[2] Corresponding author, Email address: anne-claude.camproux@u-paris.fr, Phone number: +33157278377, Postal Address: Team IsPP, 35 rue Hélène Brion, Université Paris Cité, 75205, Paris Cedex 13, FRANCE

Supplementary Section S1 – Table S1 – List of protein complexes included

This table lists the 300 protein–small molecule complexes retained for morphometric analysis. For each complex, the index, PDB identifier, protein chain, ligand three-letter code, UniProt accession number and CATH annotations are provided. This information allows full reproducibility of dataset construction. The table is available in the document “Table_S1.csv”.

Supplementary Section S2 – Pocket representation and geometric coherence

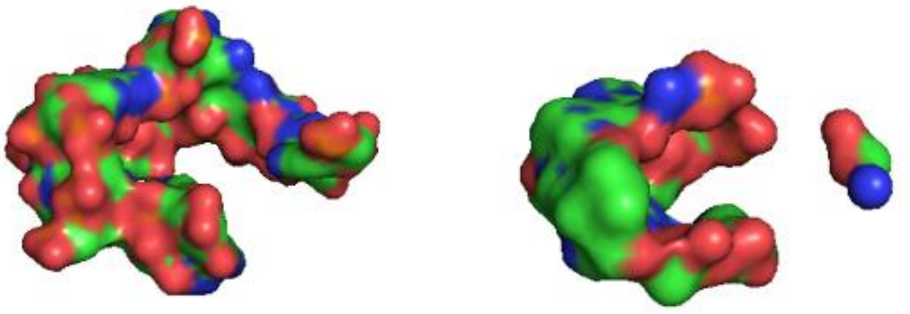

A B

Figure S2: Effect of pocket definition on the geometric coherence of the VIB binding site. Comparison of atom-level and residue-level pocket definitions for the VIB (thiamin, vitamin B1) binding site in an RNA complex PDB ID: 4NYG. (A) Residue-level pocket definition, where complete contacting residues (or nucleotides) are included when at least one heavy atom of the residue is within 6 Å of the ligand, (B) Atom-level pocket definition, where only atoms within 6 Å of the ligand are retained, leading to fragmented and discontinuous point clouds, particularly in the RNA complex.

This comparison illustrates how atom-level definitions can artificially increase apparent anisotropy and destabilize inertia-matrix-based shape descriptors, whereas residue-level definitions preserve the three-dimensional organization of the binding site and are more suitable for global morphometric analysis. All representations are based on hydrogen-free structures and the harmonized preprocessing pipeline described in Section 2.2.1. Although illustrated here for the VIB binding site, similar effects were observed across multiple RNA and protein complexes.

Supplementary Section S3 – Ligand chemical space controls

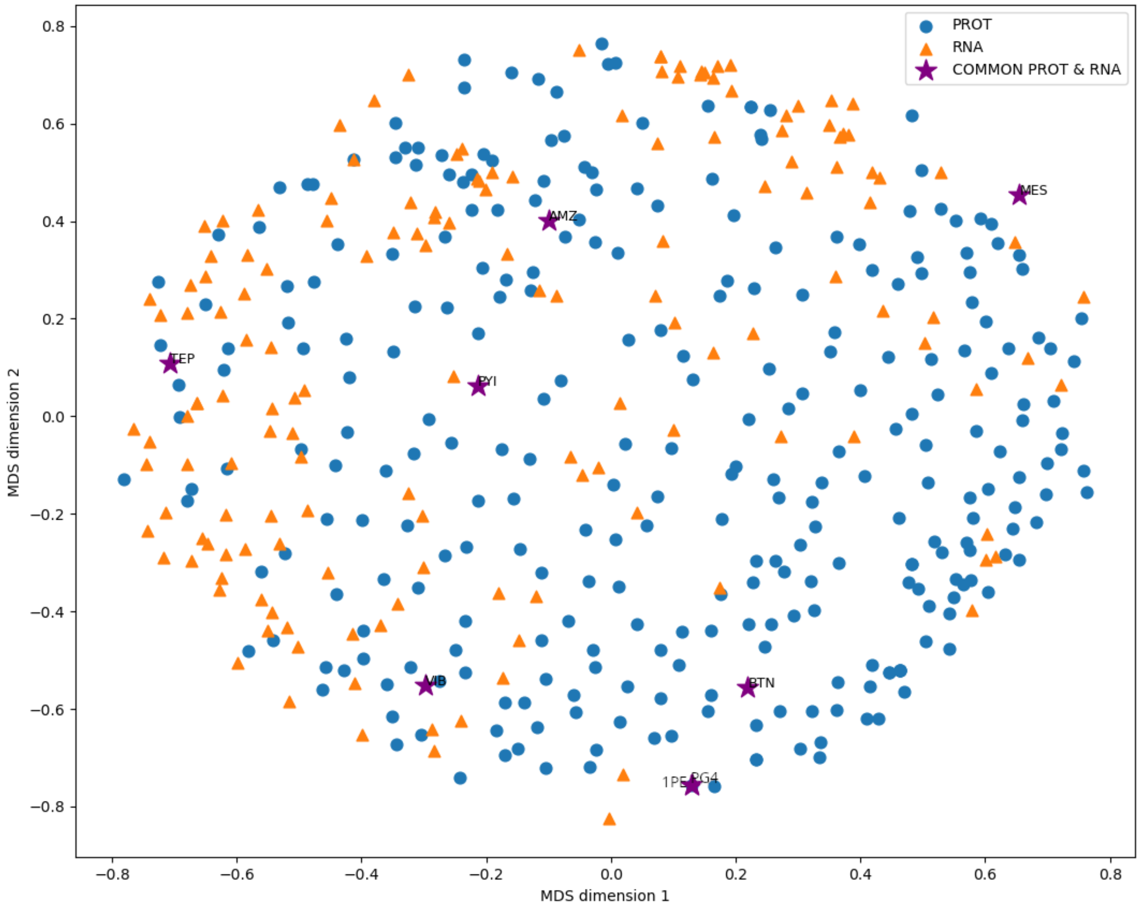

Figure S3: Overlapping ligand chemical space of RNA- and protein-binding pockets. Multidimensional scaling (MDS) representation of ligand chemical space for RNA and protein datasets based on pairwise Tanimoto similarity coefficients computed from two-dimensional Morgan fingerprints (radius = 2, 2048 bits). Each point represents a ligand bound to an RNA or a protein pocket. RNA and protein ligands occupy overlapping regions of chemical space, indicating comparable overall ligand diversity. Despite this overlap, only eight ligands are shared between the RNA and protein datasets (1PE, AMZ, BTN, MES, PG4, PYI, TEP, and VIB).

Ligand chemical space was analyzed to verify that comparative pocket shape analyses were not inadvertently conditioned by ligand chemotype or size. Ligands bound to RNA and protein pockets were represented using two-dimensional Morgan fingerprints (radius = 2, 2048 bits), and pairwise Tanimoto similarity coefficients were computed. The resulting similarity matrix was projected using multidimensional scaling (MDS). RNA- and protein-binding ligands occupy largely overlapping regions of chemical space, indicating comparable overall ligand diversity. Consistently, pairwise Tanimoto similarity distributions are highly similar for RNA-RNA, protein-protein, and RNA-protein ligand pairs, with mean similarity values in the range of ~ 0.09–0.10, indicating the absence of strong chemotype segregation between ligands associated with RNA and protein targets.

Together, these analyses confirm that RNA and protein pocket datasets are not trivially matched by ligand identity, while still sampling comparable regions of ligand chemical space. This supports the interpretation that observed pocket shape similarities and differences are not driven by ligand chemotype bias.

Supplementary Section S4 – Redundancy and dataset-dependence controls

S4.1. RNA redundancy filtering strategy

To evaluate potential dependencies arising from dataset redundancy, additional control analyses were performed. All analyses were repeated after strict redundancy filtering, retaining a single representative RNA pocket per PDB-ligand-chain combination. In the original RNA dataset (300 pockets), 254 distinct PDB entries were represented, of which 32 contributed more than one RNA pocket associated with the same ligand, yielding 65 intra-PDB pocket pairs across all combinations. This limited degree of redundancy motivated the adoption of a conservative filtering strategy, which reduced the RNA dataset from 300 to 254 pockets. To ensure balanced statistical comparisons, protein pockets were then randomly subsampled to match the RNA sample size (254 RNA vs 254 protein pockets).

S4.2. Stability of RNA-protein ratio comparisons after redundancy filtering

The full statistical analysis was repeated on the redundancy-filtered and balanced datasets (254 RNA vs 254 protein pockets). Supplementary Table S4.2 reports the comparison of RNA and protein pocket descriptors after filtering, using the same statistical framework as in the main analysis (Welch's t-tests with Holm correction across five descriptors).

Descriptor	RNA mean (SD)	PROTEIN mean (SD)	RNA n	PROTEIN n	P-value (Holm)	Cohen's d	RNA+PROTEIN median
rEV21	0.574 (0.170)	0.600 (0.165)	254	254	8.06×10^{-2}	−0.16	0.593
rEV32	0.552 (0.185)	0.605 (0.180)	254	254	1.82×10^{-3}	−0.30	0.578
rEV31	0.312 (0.130)	0.364 (0.145)	254	254	7.44×10^{-5}	−0.38	0.330
Number of atoms	253.6 (95.0)	176.9 (58.0)	254	254	2.99×10^{-25}	0.99	205.0
Radius of gyration	9.30 (1.30)	8.78 (1.05)	254	254	1.00×10^{-6}	0.42	9.05

Table S4.2: Comparison of RNA and protein binding pocket descriptors on 254 data sets. Mean values (± SD, Standard Deviation) are reported for RNA and protein pockets. Differences between macromolecular types were assessed using Welch's t-tests. P-values were adjusted for multiple testing using Holm's procedure, applied separately to shape descriptors (eigenvalue ratios rEV21, rEV32, rEV31) and to size-related descriptors (number of atoms and radius of gyration). Standardized effect sizes (Cohen's d) are reported alongside adjusted p-values to quantify the magnitude of observed differences. Global medians are also indicated.

Results obtained after redundancy filtering are highly consistent with those observed in the full dataset (300 RNA vs 300 protein pockets: Table 1 in the main text). Mean values, effect sizes, and the relative ordering of shape descriptors **(**rEV21 < rEV32 < rEV31**)** are preserved across analyses. Applying Holm's correction separately to shape and size descriptors does not alter the conclusions. Differences in secondary and global anisotropy ratios (rEV32 and rEV31) remain statistically significant with small-to-moderate effect sizes, whereas the primary anisotropy ratio rEV21 remains weakly differentiated. Size-related descriptors remain strongly discriminant. The overall interpretation of RNA-protein pocket morphometric differences is

therefore unchanged. In contrast, size-related descriptors (number of atoms and radius of gyration) display strong and stable differences in both datasets.
These results demonstrate that the observed RNA-protein morphometric differences are not driven by intra-PDB redundancy and are robust to stringent redundancy filtering.

S4.3. Archetype distributions after redundancy filtering
To further assess the robustness of the morphometric classification, the full archetype partitioning was repeated on the balanced datasets (254 RNA vs 254 protein pockets), using the same median-based morphometric decision scheme (Gaucher plane) as in the main manuscript. Medians defining the decision boundaries were re-estimated on the combined filtered dataset (RNA254 + PROT254), and pockets were reassigned to morphological archetypes accordingly.

Archetype	RNA (n)	RNA (%)	PROT (n)	PROT (%)	Total (n)	Total (%)
Anisotropic	62	24.4	49	19.3	111	21.9
Disk	79	31.1	64	25.2	143	28.1
Rod	76	29.9	67	26.4	143	28.1
Sphere	37	14.6	74	29.1	111	21.9

Table S4.3: Archetype frequency distributions. Distribution of morphological pocket archetypes in RNA and protein datasets after redundancy filtering and sample-size balancing (254 RNA vs 254 protein pockets). Counts and percentages are reported relative to each macromolecular dataset and to the total population.

A chi-square test confirmed a significant association between macromolecule type and archetype distribution after redundancy filtering and balanced sampling ($\chi^2 = 15.99$, d.f. = 3, $p < 1.13 \times 10^{-3}$).
This association reflects a higher frequency of sphere-like pockets in proteins and a higher proportion of disk-like, rod-like, and strongly anisotropic pockets in RNA under the same median-based morphometric partitioning.
Together, these sensitivity analyses demonstrate that both the continuous morphometric comparisons and the discrete archetype decomposition are robust to RNA pocket redundancy and to stringent sample-size balancing. The observed RNA-protein differences in pocket shape and archetype distributions are therefore intrinsic and reproducible, rather than driven by multiple pockets originating from the same RNA structures.

Supplementary Section S5 – Table S5 – Definition of morphometric descriptors

Ratio	Definition	Main geometric meaning	Type of descriptor	High value	Low value
rEV21 = λ2 / λ1	Relation between major and intermediate axes (x–y)	Describes axial elongation or planar symmetry	Global	Pocket more symmetric or less elongated	Strong elongation along a single axis
rEV31 = λ3 / λ1	Relation between minor and major axes (z–x)	Describes overall flattening or isotropy	Global	More spherical / isotropic shape	More flattened or elongated cavity
rEV32 = λ3 / λ2	Relation between the two smallest axes (y–z)	Describes local cross-section symmetry	Local	Circular, regular cross-section (balanced y–z)	Flattened, irregular or distorted cross-section

Table S5: Shape interpretation of eigenvalue ratios. Shape information provided by eigenvalue ratios, summarizing their definitions and the geometrical meaning in terms of high and low values.

Supplementary Section S6 – Robustness of pocket estimation

This section reports sensitivity analyses designed to assess the robustness of inertia-based shape and size descriptors with respect to pocket representation and cutoff distance.

S6.1. Effect of pocket representation: atom-level versus residue-level definition (6 Å)

Binding pockets were estimated using two alternative representations: (i) an atom-level definition including only atoms within 6 Å of the ligand, and (ii) a residue-level definition including complete contacting residues or nucleotides when at least one heavy atom lies within 6 Å of the ligand.

The residue-level definition was adopted in the main analysis to preserve geometric continuity and biological coherence, particularly for RNA binding sites. Shape descriptors (rEV21, rEV32, rEV31) and size-related descriptors (number of atoms and radius of gyration) were computed for both representations and compared using Welch's t-tests, with Holm correction applied separately to shape and size descriptors. Effect sizes were quantified using Cohen's d.

Descriptor	Atoms mean (SD)	Residues mean (SD)	P (Holm)	Cohen's d
rEV21	0.52 (0.17)	0.57 (0.17)	5.00e-4	-0.31
rEV32	0.57 (0.20)	0.55 (0.18)	2.96e-1	0.11
rEV31	0.29 (0.13)	0.31 (0.13)	2.96e-1	-0.12
Number of atoms	104.05 (40.92)	244.71 (91.76)	<1.00e-4	-1.98
Radius of gyration	7.12 (1.24)	9.25 (1.30)	<1.00e-4	-1.68

Table S6.1: Comparison of shape and size descriptors between atom-level and residue-level pocket definitions at a fixed 6 Å cutoff. Mean (± SD) values are reported for each descriptor. Statistical comparisons were performed using Welch's t-tests, with Holm correction applied separately to shape descriptors (rEV21, rEV32, rEV31) and size descriptors (number of atoms and radius of gyration). Effect sizes are reported as Cohen's d.

The quantitative results summarized in Supplementary Table S6.1 are interpreted below in terms of their impact on pocket shape versus pocket size descriptors. Shape descriptors exhibit only minor differences between representations, with small effect sizes, indicating that global pocket anisotropy is largely preserved. In contrast, size-related descriptors differ substantially, reflecting the expected increase in pocket extent when complete residues are included. These results support the use of residue-level pocket definitions for global morphometric analyses while highlighting their impact on absolute pocket size. Although a statistically significant difference is observed for rEV21, the associated effect size remains small, indicating limited practical impact on global anisotropy.

S6.2. Effect of residue-based cutoff distance (4 Å, 5 Å, and 6 Å)

To evaluate the sensitivity of pocket shape and size descriptors to the choice of cutoff distance, residue-level binding pockets were re-estimated using three cutoff values: 4 Å, 5 Å, and 6 Å. For each cutoff, shape descriptors and size-related descriptors were recomputed.

Pairwise comparisons between cutoff distances (4 Å vs 5 Å, 5 Å vs 6 Å, and 4 Å vs 6 Å) were performed using Welch's t-tests to account for unequal variances. Multiple-testing correction was applied using the Holm procedure, performed separately for shape descriptors and size-related descriptors. Standardized effect sizes were quantified using Cohen's d to assess the magnitude of differences independently of sample size.

Descriptor	Comparison A versus B	A mean (SD)	B mean (SD)	Welch t	p (raw)	p (Holm)	Cohen's d
rEV21	4 Å vs 5 Å	0.54 (0.20)	0.54 (0.18)	-0.61	0.54	5.42e-1	-0.05
	5 Å vs 6 Å	0.54 (0.18)	0.57 (0.17)	-1.95	5.20e-2	1.04e-1	-0.16
	4 Å vs 6 Å	0.54 (0.20)	0.57 (0.17)	-2.43	1.56e-2	6.24e-2	-0.20
rEV32	4 Å vs 5 Å	0.46 (0.18)	0.50 (0.18)	-2.64	8.50e-3	4.25e-2	-0.22
	5 Å vs 6 Å	0.50 (0.18)	0.55 (0.18)	-3.06	2.30e-3	1.41e-2	-0.25
	4 Å vs 6 Å	0.46 (0.18)	0.55 (0.18)	-5.78	0.00	0.00	-0.47
rEV31	4 Å vs 5 Å	0.24 (0.12)	0.27 (0.12)	-2.28	2.31e-2	6.92e-2	-0.19
	5 Å vs 6 Å	0.27 (0.12)	0.31 (0.13)	-4.18	0.00	2.00e-4	-0.34
	4 Å vs 6 Å	0.24 (0.12)	0.31 (0.13)	-6.31	0.00	0.00	-0.52
Number of atoms	4 Å vs 5 Å	164.28 (68.40)	202.00 (81.68)	-6.13	0.00	0.00	-0.50
	5 Å vs 6 Å	202.00 (81.68)	244.71 (91.76)	-6.02	0.00	0.00	-0.49
	4 Å vs 6 Å	164.28 (68.40)	244.71 (91.76)	-12.17	0.00	0.00	-0.99
Radius of gyration	4 Å vs 5 Å	8.31 (1.47)	8.77 (1.39)	-3.99	1.00e-4	1.00e-4	-0.33
	5 Å vs 6 Å	8.77 (1.39)	9.25 (1.30)	-4.34	0.00	0.00	-0.35
	4 Å vs 6 Å	8.31 (1.47)	9.25 (1.30)	-8.33	0.00	0.00	-0.68

Table S6.2: Effect of residue-based cutoff distance on pocket shape and size descriptors. Mean (± SD) values are reported for eigenvalue-ratio shape descriptors (rEV21, rEV32, rEV31) and size-related descriptors (number of atoms and radius of gyration) for RNA binding

The quantitative results summarized in Supplementary Table S6.2 provide a formal statistical assessment of descriptor stability across pocket definitions. Inertia-based shape descriptors remain largely preserved across cutoff distances, with small effect sizes, indicating robust conservation of relative pocket anisotropy. In contrast, size-related descriptors vary coherently with increasing cutoff distance, reflecting the expected expansion of pocket extent as additional residues are included. These results further support the robustness of inertia-based shape descriptors to pocket estimation parameters while highlighting the predictable sensitivity of size-related measures to spatial inclusion.

S6.3. Multivariate assessment of descriptor robustness using principal component analysis

Rather than relying solely on pairwise correlation analyses, which are limited for correlated and algebraically dependent descriptors, we assessed the robustness of pocket shape and size descriptors across pocket definitions using a multivariate principal component analysis (PCA) framework. This approach provides a global view of descriptor stability by explicitly capturing the joint structure of the descriptor space and potential systematic shifts induced by pocket definition parameters.

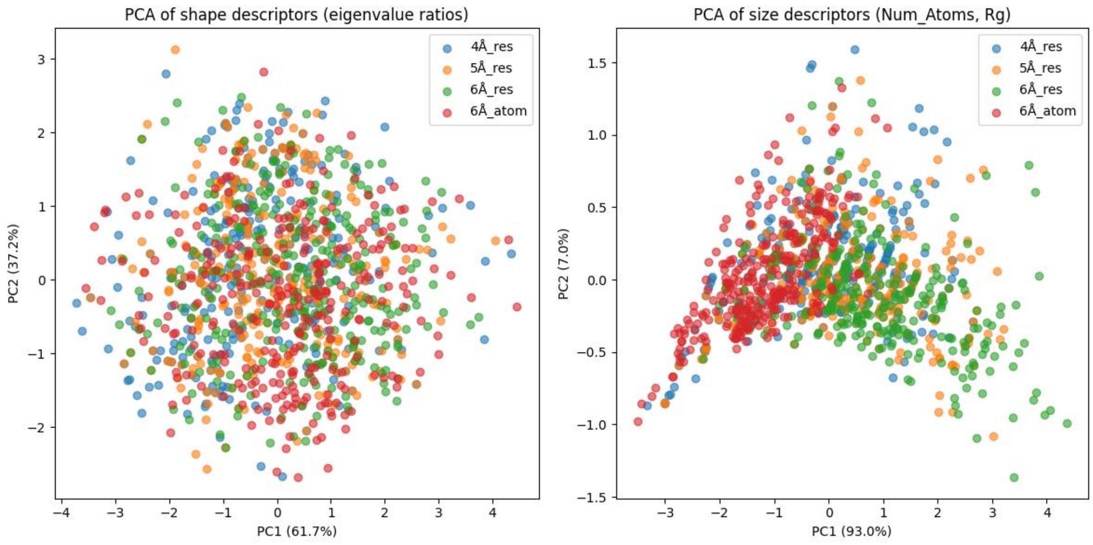

A B

Figure S6: PCA of pocket shape and size descriptors across pocket definitions.
(A) Principal component analysis (PCA) of pocket shape descriptors across pocket definitions. PCA of inertia-based shape descriptors (rEV21, rEV32, rEV31) for RNA binding pockets estimated using residue-level cutoffs of 4 Å, 5 Å, and 6 Å, as well as an atom-level definition at 6 Å. Points are colored according to pocket definition. The first two principal components account for approximately 99% of the total variance. The strong overlap between the four datasets indicates that the global organization of pocket shape space is preserved across cutoff distances and between residue- and atom-level representations, supporting the robustness of inertia-based shape descriptors to pocket definition parameters.
(B) PCA of pocket size descriptors across pocket definitions. PCA of size-related descriptors (number of atoms, Num_atoms and radius of gyration, R_g) for RNA binding pockets estimated using residue-level cutoffs of 4 Å, 5 Å, and 6 Å, as well as an atom-level definition at 6 Å. Points are colored according to pocket definition. The first principal component accounts for approximately 93% of the total variance and reflects progressive pocket expansion with increasing cutoff distance. The PCA projection displays a curved manifold rather than discrete clusters, reflecting the strong correlation and constrained, non-linear relationship between atomic content and spatial extent. This behavior indicates coherent scaling of size-related descriptors rather than instability or definition-induced artifacts.

For shape descriptors, PCA was performed on the three inertia-based eigenvalue ratios (rEV21, rEV32, rEV31) computed for RNA binding pockets estimated using residue-level cutoffs of 4 Å, 5 Å, and 6 Å, as well as an atom-level definition at 6 Å. The first two principal components account for approximately 99% of the total variance. As shown in Supplementary Figure S6A the strong overlap between point clouds corresponding to the different pocket definitions indicates that the global organization of the shape space is preserved across cutoff distances and between residue- and atom-level representations. No systematic displacement or separation associated with pocket definition is observed, supporting the robustness of inertia-based shape descriptors to these parameters.

For size-related descriptors, PCA was applied to the number of atoms and the radius of gyration (R_g) computed for the same pocket definitions. In this case, the first principal component captures approximately 93% of the total variance and primarily reflects progressive pocket expansion with increasing cutoff distance. The resulting projection displays a curved manifold rather than discrete clusters (Supplementary Figure S6B), reflecting the strong correlation and constrained, non-linear relationship between atomic content and spatial extent. This behavior is expected and indicates coherent scaling of size-related descriptors rather than instability or definition-induced artifacts.

Together, these multivariate analyses complement the univariate statistical comparisons reported in Supplementary S4.1 and S4.2. They confirm that inertia-based shape descriptors exhibit high stability across pocket representations and cutoff distances, while size-related descriptors vary in a predictable and physically meaningful manner. PCA thus provides an integrated and visually interpretable assessment of descriptor robustness that is better suited than pairwise correlation analyses for evaluating descriptor behavior in the presence of correlated and algebraically dependent variables.

Supplementary Section S7 – Sensitivity of inertia-based pocket descriptors to hydrogen inclusion

S7.1. Rationale and analysis design

Although all primary analyses were performed on hydrogen-free structures following the harmonized preprocessing pipeline described in Section 2.2.1, a subset of deposited RNA and protein structures originally contained explicit hydrogen atoms. To quantitatively assess whether hydrogen inclusion could affect inertia-based pocket descriptors, a dedicated sensitivity analysis was conducted.

The analysis was performed on a subset of complexes available in both structural representations, comprising 20 RNA pockets and 11 protein pockets. For each complex, binding pockets were independently estimated from the hydrogen-free and hydrogen-included structures using the same ligand-centered proximity procedure described in the Methods. In practice, the resulting pockets involved the same set of residues, with the only difference being the presence of hydrogen atoms in the atomic representation.

Eigenvalue ratios (rEV21, rEV32, rEV31) and size-related descriptors (number of pocket atoms) were then recomputed for hydrogen-free (noH) and hydrogen-included (withH) representations for both RNA and protein pockets.

S7.2. Pocket-wise stability of eigenvalue ratios

Across the combined RNA and protein datasets, eigenvalue ratios exhibit extremely high correlations between hydrogen-free and hydrogen-included representations (Supplementary Figure S7), with $r = 0.988$ ($p = 2.72 \times 10$–25) for rEV21, $r = 0.978$ ($p = 3.21 \times 10^{-21}$) for rEV32, and $r = 0.978$ ($p = 3.21 \times 10^{-21}$) for rEV31, and $r = 0.988$ ($p = 7.36 \times 10^{-25}$) for rEV31. RNA and protein pockets follow the same diagonal trend, with no visible macromolecule-specific deviation. These results indicate that relative pocket anisotropy is preserved upon hydrogen inclusion when residue membership is held constant.

System-specific analyses further confirm this stability. For RNA pockets, correlations between noH and withH eigenvalue ratios exceed $r \geq 0.995$ for all three descriptors. For protein pockets, correlations remain very high ($r \approx 0.96$–0.98). Together, these results demonstrate strong morphometric consistency across atomic representations.

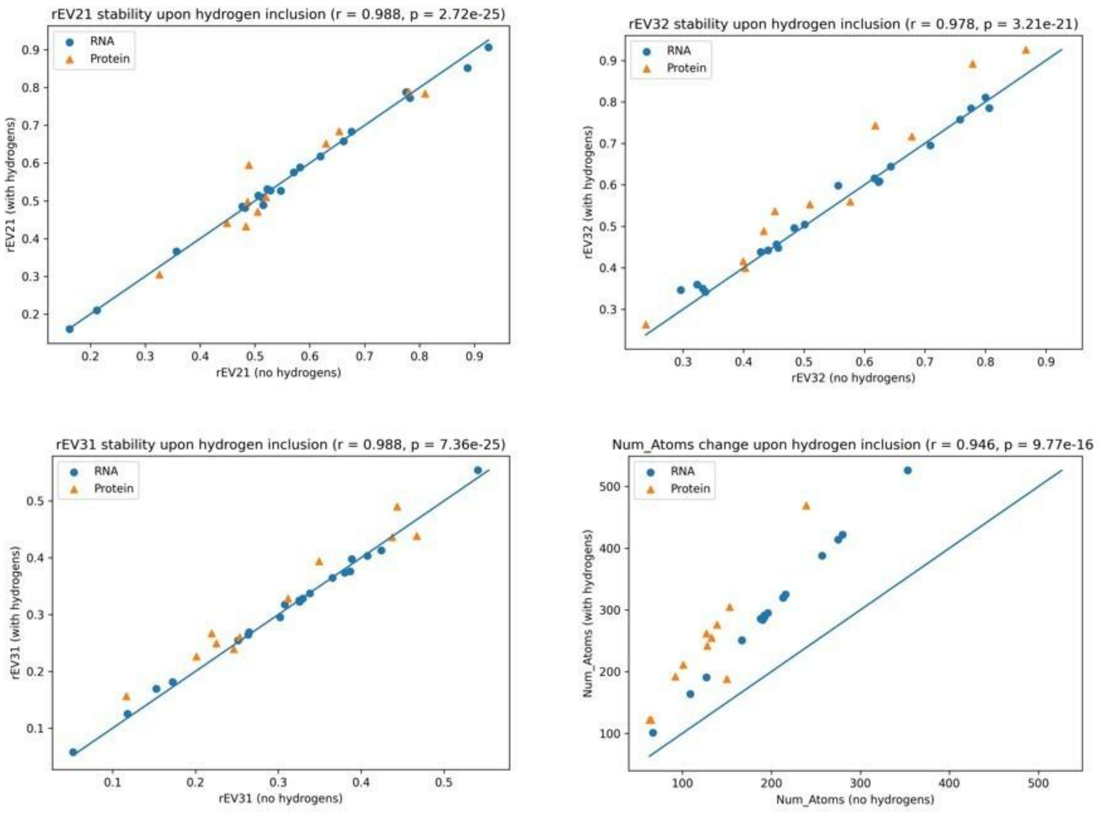

Figure S7: Stability of inertia-based pocket descriptors upon hydrogen inclusion. Scatter plots comparing hydrogen-free (noH) and hydrogen-included (withH) representations for eigenvalue-ratio descriptors (rEV21, rEV32, rEV31) and Num-Atoms. 20 RNA pockets are shown as circles and 11 protein pockets as triangles. Pearson correlation coefficients (r) and associated p-values are computed across the combined RNA and protein datasets and reported in each panel. Eigenvalue ratios exhibit extremely high correlations ($r > 0.96$), whereas hydrogen inclusion primarily increases the number of atoms without inducing systematic distortion in relative pocket anisotropy.

System	Descriptor	noH Mean (SD)	withH Mean (SD)	Pearson r	p-value
RNA	Eigval 1	38.904 (7.484)	39.598 (7.293)	0.998	1.27e-22
RNA	Eigval 2	21.704 (8.433)	21.989 (8.403)	0.999	2.26e-27
RNA	Eigval 3	11.745 (4.879)	12.026 (4.867)	0.998	7.41e-24
RNA	rEV21	0.565 (0.193)	0.562 (0.189)	0.998	6.18e-23
RNA	rEV32	0.548 (0.167)	0.555 (0.158)	0.995	2.31e-19
RNA	rEV31	0.305 (0.115)	0.307 (0.112)	0.998	5.59e-23
RNA	Num_Atoms	198.60 (62.16)	298.65 (92.97)	1.000	1.55e-33
PROT	Eigval 1	33.946 (7.883)	33.645 (8.086)	0.984	4.33e-08
PROT	Eigval 2	19.398 (8.799)	19.348 (8.934)	0.992	1.60e-09
PROT	Eigval 3	10.434 (5.766)	10.990 (5.441)	0.995	2.04e-10
PROT	rEV21	0.557 (0.146)	0.560 (0.154)	0.962	2.35e-06
PROT	rEV32	0.541 (0.184)	0.590 (0.208)	0.981	1.07e-07
PROT	rEV31	0.297 (0.114)	0.317 (0.107)	0.977	2.64e-07
PROT	Num_Atoms	126.364 (48.751)	240.364 (96.110)	0.938	1.91e-05

Table S7.2: Comparison of inertia-based and size-related pocket descriptors between hydrogen-free (noH) and hydrogen-included (withH) representations. Mean values (± SD) are reported separately for RNA and protein pockets. Pearson correlation coefficients (r) and associated p-values quantify the stability of each descriptor across representations. Eigenvalue-ratio descriptors (rEV21, rEV32, rEV31) remain highly

correlated between noH and withH representations, whereas the number of atoms increases substantially as expected. These results demonstrate the robustness of inertia-based morphometric descriptors to hydrogen inclusion under fixed pocket membership.

S7.3. Median stability across representations

Median eigenvalue ratios remained highly stable upon hydrogen inclusion. For RNA pockets (n = 20), median rEV21 changed from 0.538 to 0.529 and median rEV32 from 0.529 to 0.552; for protein pockets (n = 11), median rEV21 changed from 0.505 to 0.510 and median rEV32 from 0.510 to 0.553.

While a moderate displacement is observed for protein rEV32, this shift does not induce a systematic reorganization of the morphometric landscape and remains consistent with the high correlation structure observed at the pocket level. Median shifts remain small in magnitude relative to the global variance of the datasets.

These results indicate that hydrogen inclusion may slightly affect absolute inertia magnitudes but does not reorganize the relative anisotropy structure captured by eigenvalue ratios.

S7.4. Independence between hydrogen-induced atom count increase and anisotropy variation

As expected, transitioning from hydrogen-free to hydrogen-included pocket representations substantially increases the absolute number of atoms per pocket. In 20 RNA pockets, the average atom count rises from 198.6 in the hydrogen-free representation to 298.7 when hydrogen atoms are included, whereas in 11 protein pockets the mean atom count increases from 126.4 to 240.4.

Across the combined dataset of 31 pockets (20 RNA and 11 protein), correlation analyses between hydrogen-induced changes in atom count (ΔNum_Atoms) and changes in eigenvalue ratios (ΔrEV) reveal no significant linear association, with $r = -0.144$ ($p = 0.440$) for ΔrEV21, $r = 0.125$ ($p = 0.503$) for ΔrEV32, and $r = -0.174$ ($p = 0.348$) for ΔrEV31.

Thus, increases in pocket atom count due to hydrogen inclusion are not associated with systematic changes in anisotropy descriptors. This absence of association confirms that inertia-based shape descriptors capture global spatial organization rather than atom-count–driven scaling effects.

Taken together, these analyses demonstrate that inertia-based eigenvalue ratios are robust to hydrogen inclusion when pocket membership is held constant. Hydrogen atoms increase pocket size descriptors as expected but do not induce systematic reorganization of principal inertia axes or relative anisotropy regimes.

These results quantitatively support the methodological choice of heavy-atom representations in the primary analyses and confirm that hydrogen removal does not bias the global morphometric conclusions reported in the main manuscript. These results confirm that inertia-based eigenvalue ratios are robust to hydrogen inclusion and therefore support the use of heavy-atom representations in the primary analyses.

Supplementary Section S8 – Conserved pocket shape but divergent pocket size across RNA and protein archetypes

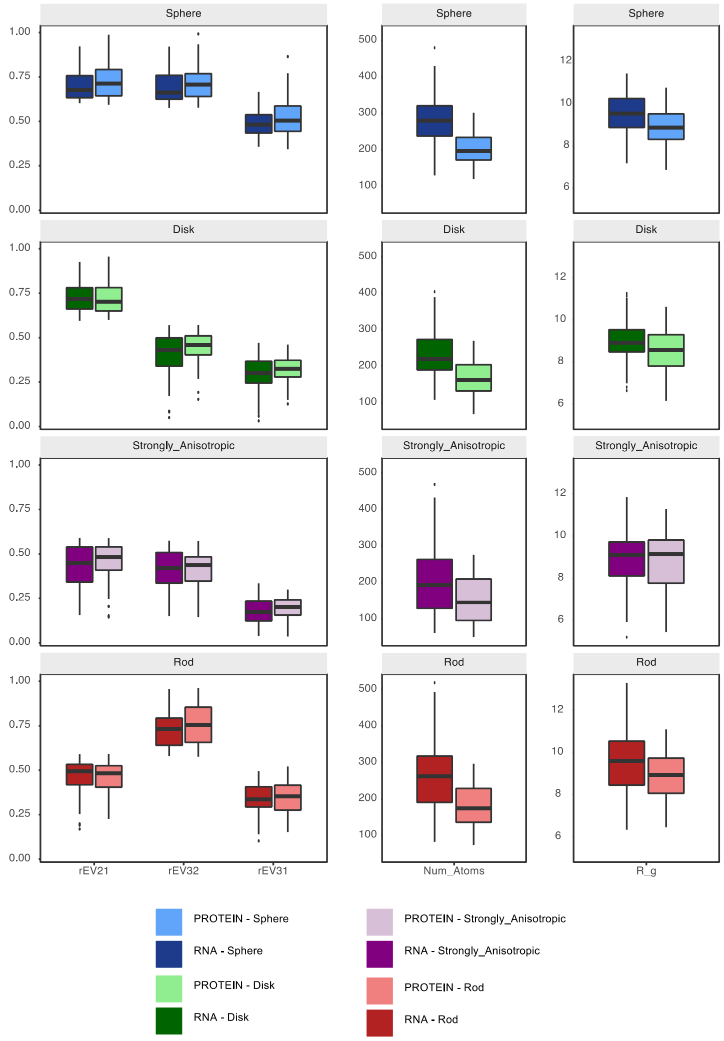

Figure S8: Conserved pocket shape but divergent pocket size across RNA and protein archetypes. Distribution of eigenvalue-ratio shape descriptors and pocket size for RNA and protein binding pockets stratified by morphological archetypes. For each archetype (sphere-like, disk-like, rod-like, and strongly anisotropic), boxplots show the distributions of the three scale-invariant shape descriptors rEV21 (global elongation), rEV32 (cross-sectional symmetry), and rEV31 (derived overall anisotropy), together with the number of atoms (Num_Atoms) and radius of gyration (R_g) which reflects absolute pocket size. Eigenvalue ratios quantify relative pocket anisotropy on a normalized scale independent of pocket size, whereas the number of atoms and number of atoms capture absolute pocket extent.

Eigenvalue ratios are defined on a normalized [0,1] scale and capture relative pocket anisotropy independently of pocket size, whereas the number of atoms defining each pocket and number of atoms quantify absolute pocket extent. Within each archetype, RNA and protein pockets display substantial overlap in eigenvalue-ratio distributions, consistent with the absence of systematic multivariate shape differences between macromolecular types when geometry is controlled for. In contrast, number of atoms and number of atoms exhibits a marked and systematic shift toward larger values for RNA pockets across all archetypes. This figure illustrates the decoupling between conserved relative pocket shape and divergent absolute pocket size between RNA and protein binding sites.

Supplementary Section S9 – Multivariate and univariate statistical analyses within morphological archetypes

This section provides the statistical analyses supporting the absence of systematic RNA-protein shape differences within morphological archetypes and the presence of consistent size-related differences reported in Section 3.4.2 of the main text.

S9.1. Multivariate assessment of pocket shape within archetypes (MANOVA)

To formally assess whether macromolecular identity (RNA versus protein) introduces residual shape differences within each morphological archetype, multivariate analyses were performed on the two independent inertia-based eigenvalue ratios rEV21 and rEV32. These descriptors capture complementary aspects of global pocket anisotropy and jointly define the primary shape space used for archetype classification.

Multivariate differences between RNA and protein pockets within each morphological archetype were assessed using MANOVA (Pillai's trace), with macromolecular type as the explanatory factor and rEV21 and rEV32 as dependent variables.

Archetype	Pillai	F	df1	df2	p-value
Sphere	0.0273	1.85	2	132	0.161
Disk	0.0334	2.80	2	162	0.064
Strongly anisotropic	0.0156	1.05	2	132	0.353
Rod	0.0191	1.58	2	162	0.210

Table S9.1: Multivariate comparison of RNA and protein pocket shapes within archetypes (MANOVA). Multivariate comparison of RNA and protein binding pocket shapes within each morphological archetype using MANOVA (Pillai's trace). Macromolecular type (RNA versus protein) was used as the explanatory factor, and the two independent inertia-based eigenvalue ratios rEV21 and rEV32 were used as dependent variables. For each archetype, Pillai's trace statistic, F value, degrees of freedom, and associated p-value are reported.

This analysis assesses whether macromolecular identity introduces systematic differences in relative pocket anisotropy within archetypes. Across all four archetypes, no statistically significant multivariate RNA-protein shape differences were detected. A marginal trend was observed for disk-like pockets, but this effect does not reach conventional significance thresholds and is not consistently supported by univariate analyses.

S9.2. Univariate analyses of pocket shape descriptors within archetypes

Complementary univariate comparisons were performed for the three eigenvalue-ratio shape descriptors within each morphological archetype. Welch's t-tests were used to account for unequal variances. False discovery rate (FDR) correction was applied to the two independent ratios rEV21 and rEV32 within each archetype. The derived ratio rEV31, being algebraically dependent on rEV21 and rEV32, was analyzed separately as a univariate descriptor and is reported without multiple-testing correction.

Archetype	Ratio	RNA mean	PROTEIN mean	p (FDR)	Cohen's d
Sphere	rEV21	0.70	0.72	0.20	-0.23
	rEV32	0.69	0.72	0.20	-0.26
	rEV31	0.49	0.52	-	-0.29
Disk	rEV21	0.73	0.73	0.95	0.01
	rEV32	0.41	0.45	0.03	-0.37
	rEV31	0.30	0.32	-	-0.31
Strongly anisotropic	rEV21	0.43	0.46	0.31	-0.24
	rEV32	0.42	0.41	0.85	0.03
	rEV31	0.18	0.19	-	-0.17
Rod	rEV21	0.46	0.46	0.97	0.01
	rEV32	0.73	0.76	0.16	-0.28
	rEV31	0.34	0.35	-	-0.16

Table S9.2: Univariate comparison of RNA and protein pocket shape descriptors within archetypes. Univariate comparison of RNA and protein binding pockets within each morphological archetype across inertia-based shape descriptors. Mean values are reported for RNA and protein pockets. Differences were assessed using Welch's t-tests to account for unequal variances. False discovery rate (FDR) correction was applied to the two independent eigenvalue ratios rEV21 and rEV32 within each archetype. The derived ratio rEV31, being algebraically dependent on rEV21 and rEV32, was analyzed separately as a univariate descriptor and is therefore reported without multiple-testing correction. Multivariate differences in shape descriptors were assessed independently using MANOVA.

These analyses confirm the absence of systematic RNA-protein differences in relative pocket anisotropy within archetypes, consistent with the MANOVA results.

S9.3. Univariate analyses of pocket size descriptors within archetypes

Size-related descriptors number of atoms and radius of gyration were analyzed independently within each archetype using Welch's t-tests. FDR correction was applied across the two size descriptors. Effect sizes are reported as Cohen's d.

Archetype	Descriptor	RNA mean	PROTEIN mean	p (FDR)	Cohen's d
Sphere	Number of atoms	214.20	152.50	2.8e-08	0.96
	Radius of gyration	7.12	6.63	1.96-02	0.41
Disk	Number of atoms	198.3	144.00	4.4e-09	0.87
	Radius of gyration	7.01	6.55	2.6e-02	0.36
Strongly anisotropic	Number of atoms	191.50	133.40	9.6e-06	0.72
	Radius of gyration	6.87	6.81	0.63	0.08
Rod	Number of atoms	205.10	139.10	6.8e-08	0.82
	Radius of gyration	7.08	6.74	5.2e-02	0.32

Table S9.3: Univariate comparison of RNA and protein pocket size descriptors within archetypes. Univariate comparison of RNA and protein binding pockets within each morphological archetype across size-related descriptors. Mean values are reported for RNA and protein pockets. Differences were assessed using Welch's t-tests to account for unequal variances. Size-related descriptors: number of atoms and radius of gyration were analyzed independently using Welch's t-tests, with FDR correction applied across these two size descriptors. Effect sizes are reported as Cohen's d.

Across all archetypes, RNA pockets involve significantly larger structural environments than protein pockets, with large effect sizes for number of atoms and small to moderate effect sizes for radius of gyration, depending on the archetype.

S9.4. Interpretation and scope

Taken together, these analyses demonstrate that when binding pockets are stratified by morphological archetypes, RNA and protein pockets exhibit highly comparable relative shape organization, as captured by inertia-based eigenvalue ratios. The absence of significant multivariate effects indicates that macromolecular identity does not introduce a systematic shape divergence within archetypes.

In contrast, size-related descriptors reveal consistent RNA-protein differences across archetypes, indicating that RNA binding pockets typically involve larger structural environments. This dissociation confirms that eigenvalue ratios primarily encode relative pocket geometry, whereas absolute size descriptors capture orthogonal information related to macromolecular composition and structural context. These supplementary analyses provide the statistical foundation for the conclusions reported in the main text regarding conserved pocket shape organization and divergent pocket size between RNA and protein binding sites.

Supplementary Section S10 – Association between Morphometric Archetypes and Ligand Physicochemical Properties

Here, we performed exploratory descriptive analyses to assess whether morphometric archetypes are associated with ligand chemotype characteristics. The following ligand descriptors were considered: molecular weight (MW), quantitative estimate of drug-likeness (QED), lipophilicity (ALOGP), polar surface area (PSA), and aromatic ring count (AROM). Together, these descriptors capture complementary aspects of molecular size, polarity, lipophilicity, and structural complexity. For each system (RNA and protein), these descriptors were compared across the four morphometric archetypes (Disk, Rod, Sphere, and Strongly Anisotropic).

The distributions of ligand descriptors across archetypes are shown in Figure S10, and corresponding quantitative summary statistics (mean ± SD) are provided in Table S10. As illustrated in Figure S10, descriptor distributions largely overlap across archetypes in both RNA and protein systems. Although moderate shifts in central tendency are observed for some descriptors, no morphometric class exhibits a clearly distinct physicochemical profile.

Table S10 provides quantitative summary statistics corresponding to the distributions shown in Figure S10. For each descriptor and archetype, mean (SD) values are reported, and column headers indicate the number of pocket–ligand complexes and unique ligands (pockets/unique ligands), allowing assessment of sample size balance. Because some ligands occur in multiple pocket–ligand complexes, analyses were conducted at the complex level. The number of unique ligands per archetype is therefore reported to provide transparency regarding potential redundancy. The "Global" category represents the complete ligand set independently of archetype. These numerical summaries corroborate the substantial distributional overlap observed across archetypes.

To formally assess the global effect of archetype on ligand physicochemical properties, a multivariate analysis of variance (MANOVA) using Pillai's trace was conducted for the descriptor vector [MW, QED, ALOGP, PSA, AROM]. Pillai's trace was selected for its robustness to unequal sample sizes and moderate deviations from covariance homogeneity. The analysis assumes approximate independence of observations at the complex level, approximate multivariate normality, and homogeneity of covariance matrices across groups.

A statistically significant multivariate effect of archetype was detected in both systems (RNA: Pillai's trace = 0.195, $p < 0.001$; protein: Pillai's trace = 0.110, $p = 0.0045$). However, descriptor distributions remained largely overlapping across archetypes in both RNA and protein systems (Figure S10; Table S10), and effect magnitudes remained small to moderate, indicating limited practical separation between archetypes. No morphometric class was associated with a distinct or exclusive chemotype profile.

Together, these results indicate that morphometric classes are not trivially defined by ligand chemistry and support weak graded modulation of ligand physicochemical properties by pocket geometry rather than discrete chemical specialization.

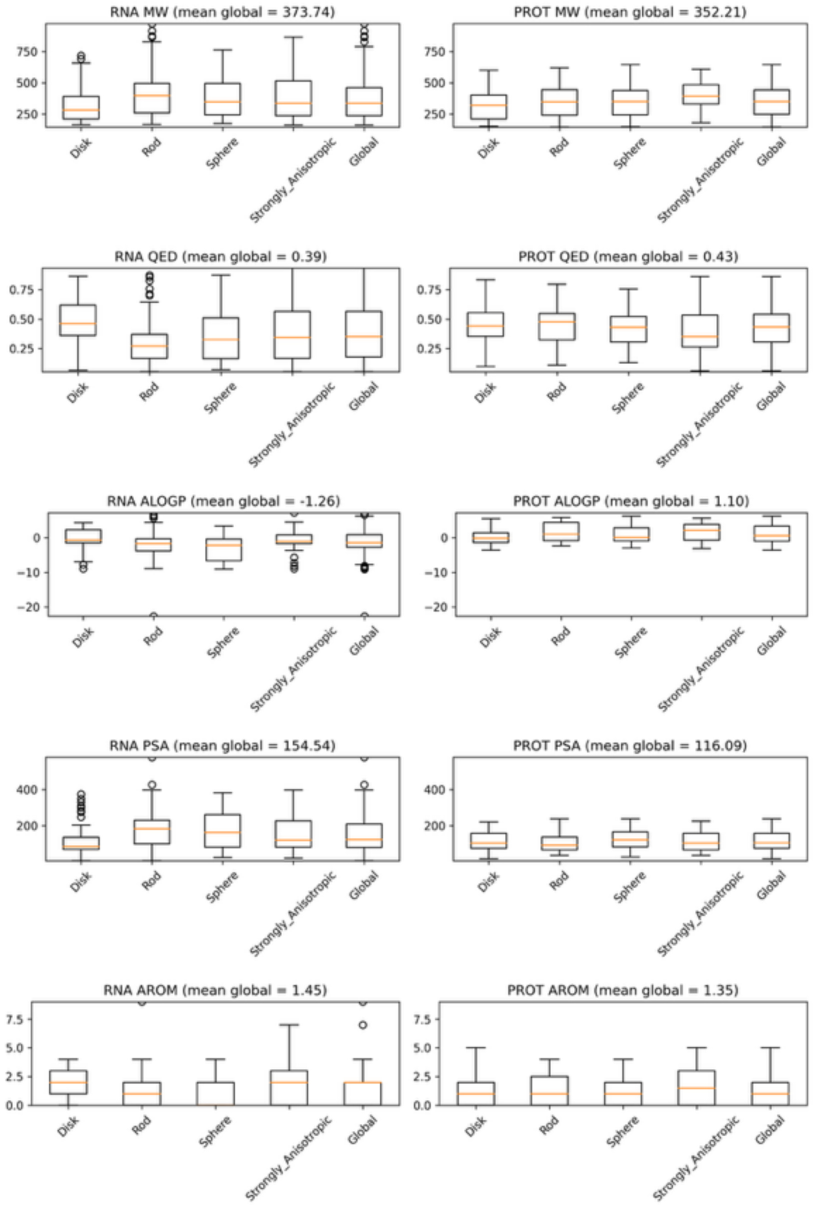

Figure S10. Ligand physicochemical descriptors across morphometric archetypes in RNA and protein systems. Boxplots display MW, QED, ALOGP, PSA, and AROM for the four morphometric archetypes (Disk, Rod, Sphere, Strongly Anisotropic). RNA (left panels) and protein (right panels) are shown using identical y-axis scales for each descriptor.

System	Descriptor	Disk (#91/#63)	Rod (#85/#65)	Sphere (#49/#35)	Strongly_Anisotropic (#74/#56)	Global
RNA	MW	311.21 (124.40)	420.75 (187.79)	392.52 (163.36)	384.20 (175.89)	373.74 (168.53)
RNA	QED	0.48 (0.19)	0.31 (0.20)	0.36 (0.22)	0.38 (0.23)	0.39 (0.22)
RNA	ALOGP	-0.01 (2.69)	-2.11 (4.08)	-2.87 (3.58)	-0.74 (2.95)	-1.26 (3.50)
RNA	PSA	111.24 (78.28)	185.84 (100.93)	180.22 (105.25)	154.84 (99.26)	154.54 (99.40)
RNA	AROM	1.79 (1.26)	1.19 (1.50)	0.84 (1.11)	1.76 (1.43)	1.45 (1.40)
**System**	**Descriptor**	**Disk (#74/#74)**	**Rod (#79/#79)**	**Sphere (#86/#86)**	**Strongly_Anisotropic (#60/#60)**	**Global**
PROT	MW	326.76 (126.19)	342.63 (129.52)	349.88 (124.35)	399.55 (103.34)	352.21 (124.31)
PROT	QED	0.45 (0.17)	0.45 (0.17)	0.42 (0.16)	0.40 (0.19)	0.43 (0.17)
PROT	ALOGP	0.43 (2.36)	1.52 (2.65)	0.84 (2.39)	1.74 (2.56)	1.10 (2.53)
PROT	PSA	114.67 (51.52)	107.05 (51.80)	126.05 (51.26)	115.46 (51.45)	116.09 (51.74)
PROT	AROM	1.14 (1.21)	1.44 (1.34)	1.31 (1.26)	1.55 (1.48)	1.35 (1.32)

Table S10. Mean (SD) of ligand physicochemical descriptors across morphometric archetypes in RNA and protein systems. Mean (SD) of ligand physicochemical descriptors across morphometric archetypes in RNA and protein systems. For each system, descriptor values are reported as mean (standard deviation) within each archetype. Column headers indicate the number of pocket–ligand complexes and the number of unique ligands per archetype (pockets/unique ligands). These quantitative summaries support the distributional overlap observed in Figure S10.

Supplementary Data S11 - RNA structural class distribution across morphometric archetypes

Supplementary Table S11 reports the distribution of RNA binding pockets across morphometric archetypes as a function of RNA structural class. Percentages are reported relative to each archetype (row-wise) to assess whether specific RNA families disproportionately populate individual pocket shapes.

Archetype	Regulatory RNA	Riboswitch	Structured functional RNA	mRNA	rRNA	Global
Sphere	1 (2.0%)	15 (30.6%)	16 (32.7%)	1 (2.0%)	16 (32.7%)	49 (100%)
Disk	2 (2.2%)	38 (41.8%)	45 (49.5%)	1 (1.1%)	5 (5.5%)	91 (100%)
Strongly anisotropic	6 (8.0%)	33 (44.0%)	30 (40.0%)	1 (1.3%)	5 (6.7%)	75 (100%)
Rod	11 (12.9%)	22 (25.9%)	24 (28.2%)	2 (2.4%)	26 (30.6%)	85 (100%)
Total	20 (6.7%)	108 (36.0%)	115 (38.3%)	5 (1.7%)	52 (17.3%)	300 (100%)

Table S11: Distribution of RNA structural types across morphometric pocket archetypes. Contingency table reporting the number and percentage of RNA binding pockets assigned to each morphometric archetype as a function of RNA structural class. Percentages are reported relatively to each archetype (row-wise). RNA structural classes were defined as regulatory RNAs, riboswitches, structured functional RNAs, mRNAs, and ribosomal RNAs (rRNAs). The global distribution of archetypes in the RNA dataset is reported for reference.

Ribosomal RNAs represent 17.3% of the RNA dataset and are distributed across all four archetypes. Notably, sphere-like pockets are not dominated by rRNAs, but instead arise from a heterogeneous set of RNA classes, including riboswitches and structured functional RNAs. Similarly, no single RNA structural class exclusively populates any morphometric archetype.

A chi-square test of independence ($\chi^2 = 48.20$, df = 12, $p < 2.9 \times 10^{-6}$) indicates a statistically significant association between RNA structural class and pocket archetype. However, this association reflects shifts in relative frequencies rather than one-to-one correspondence between RNA classes and morphometric regimes. Importantly, the observed RNA-protein differences in archetype occupancy cannot be attributed to over-representation of ribosomal RNAs or any single RNA family.

These results support the interpretation that morphometric archetypes capture generic geometric organizations recurring across diverse RNA architectures, rather than class-specific pocket geometries.
