# Supplementary figures and images for: Eigenvalue Ratios Reveal Shared Binding Pocket Shapes in RNA and Protein Structures
Source: Comput Struct Biotechnol J. 2026 Apr 20;35(1):0022. doi: 10.34133/csbj.0022 (PMC13094404; doi:10.34133/csbj.0022)

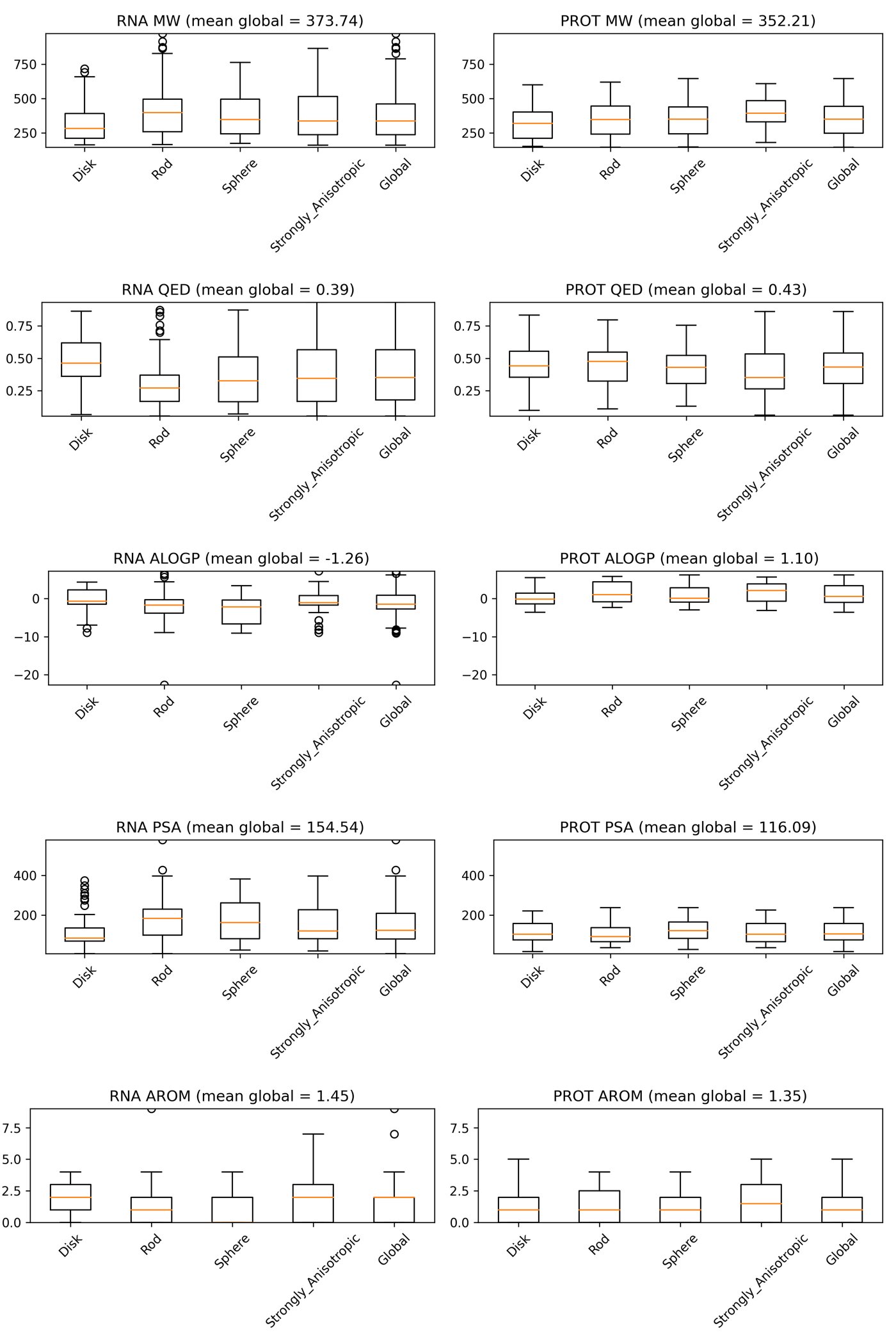

Supplement: Supplementary 1 — Section S1: Table S1 Section S2: Fig. S2 Section S3: Fig. S3 Section S4: Tables S4.2 and S4.3 Section S5: Table S5 Section S6: Tables S6.1 and S6.2 and Fig. S6 Section S7: Fig. S7 and Table S7.2 Section S8: Fig. S8 Section S9: Tables S9.1, S9.2, and S9.3 Section S10: Fig. S10 and Table S10 Data S11: Table S11 [file csbj.0022.f1.zip › FigureS10.jpg]

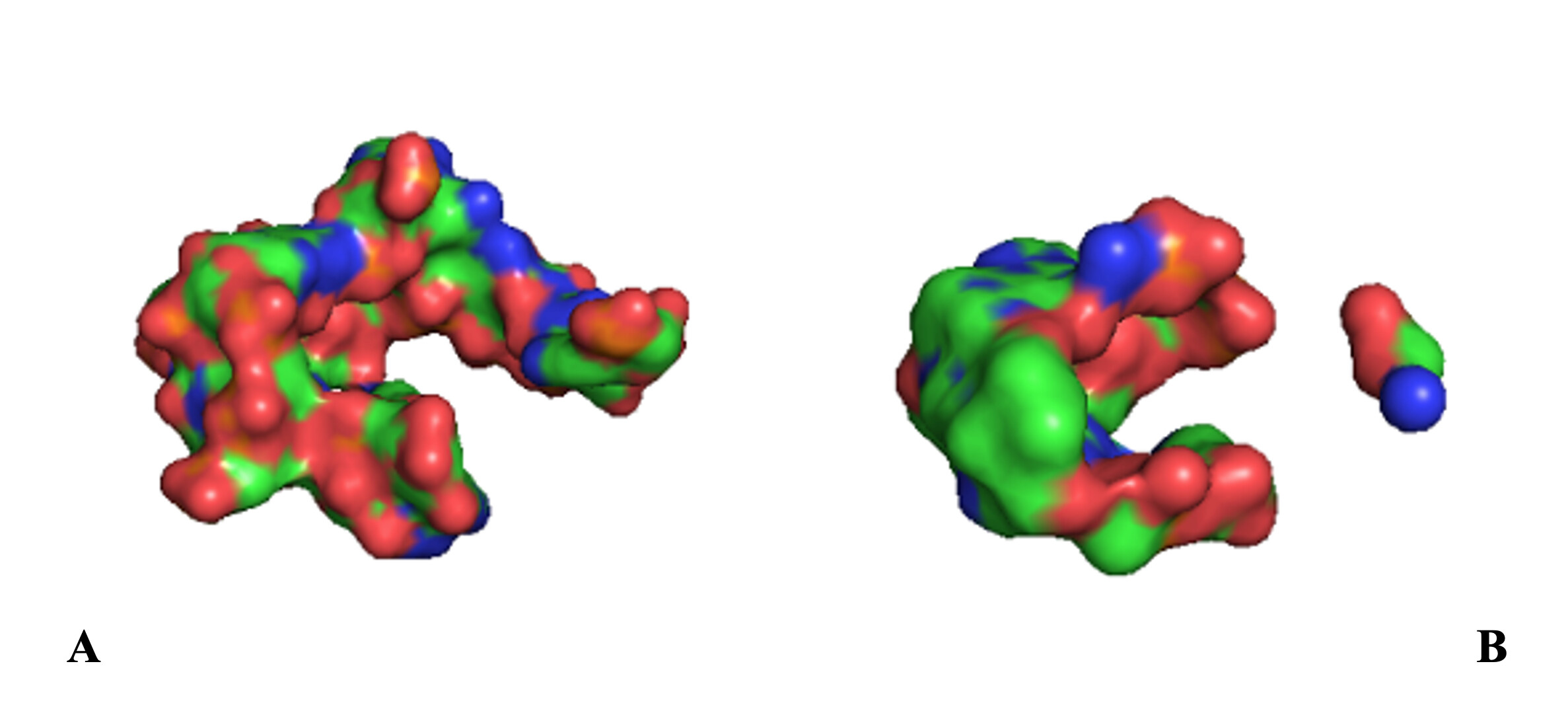

Supplement: Supplementary 1 — Section S1: Table S1 Section S2: Fig. S2 Section S3: Fig. S3 Section S4: Tables S4.2 and S4.3 Section S5: Table S5 Section S6: Tables S6.1 and S6.2 and Fig. S6 Section S7: Fig. S7 and Table S7.2 Section S8: Fig. S8 Section S9: Tables S9.1, S9.2, and S9.3 Section S10: Fig. S10 and Table S10 Data S11: Table S11 [file csbj.0022.f1.zip › FigureS2.jpg]

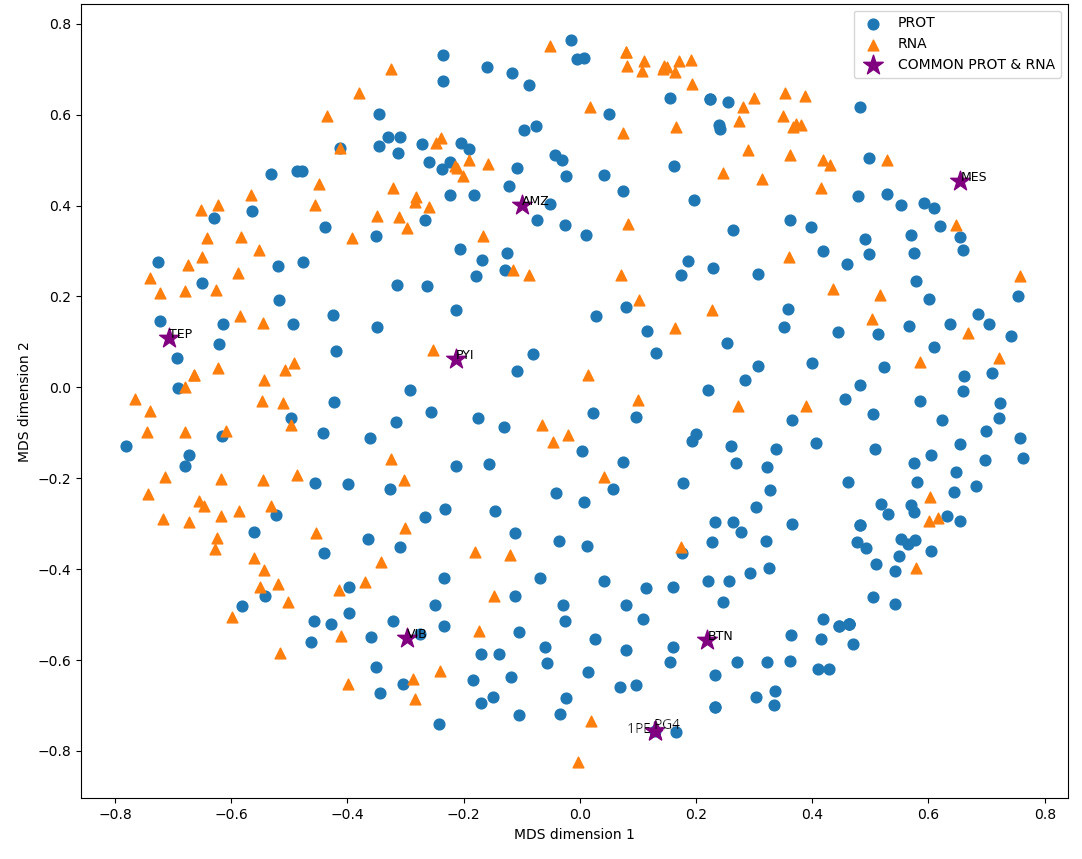

Supplement: Supplementary 1 — Section S1: Table S1 Section S2: Fig. S2 Section S3: Fig. S3 Section S4: Tables S4.2 and S4.3 Section S5: Table S5 Section S6: Tables S6.1 and S6.2 and Fig. S6 Section S7: Fig. S7 and Table S7.2 Section S8: Fig. S8 Section S9: Tables S9.1, S9.2, and S9.3 Section S10: Fig. S10 and Table S10 Data S11: Table S11 [file csbj.0022.f1.zip › FigureS3.jpg]

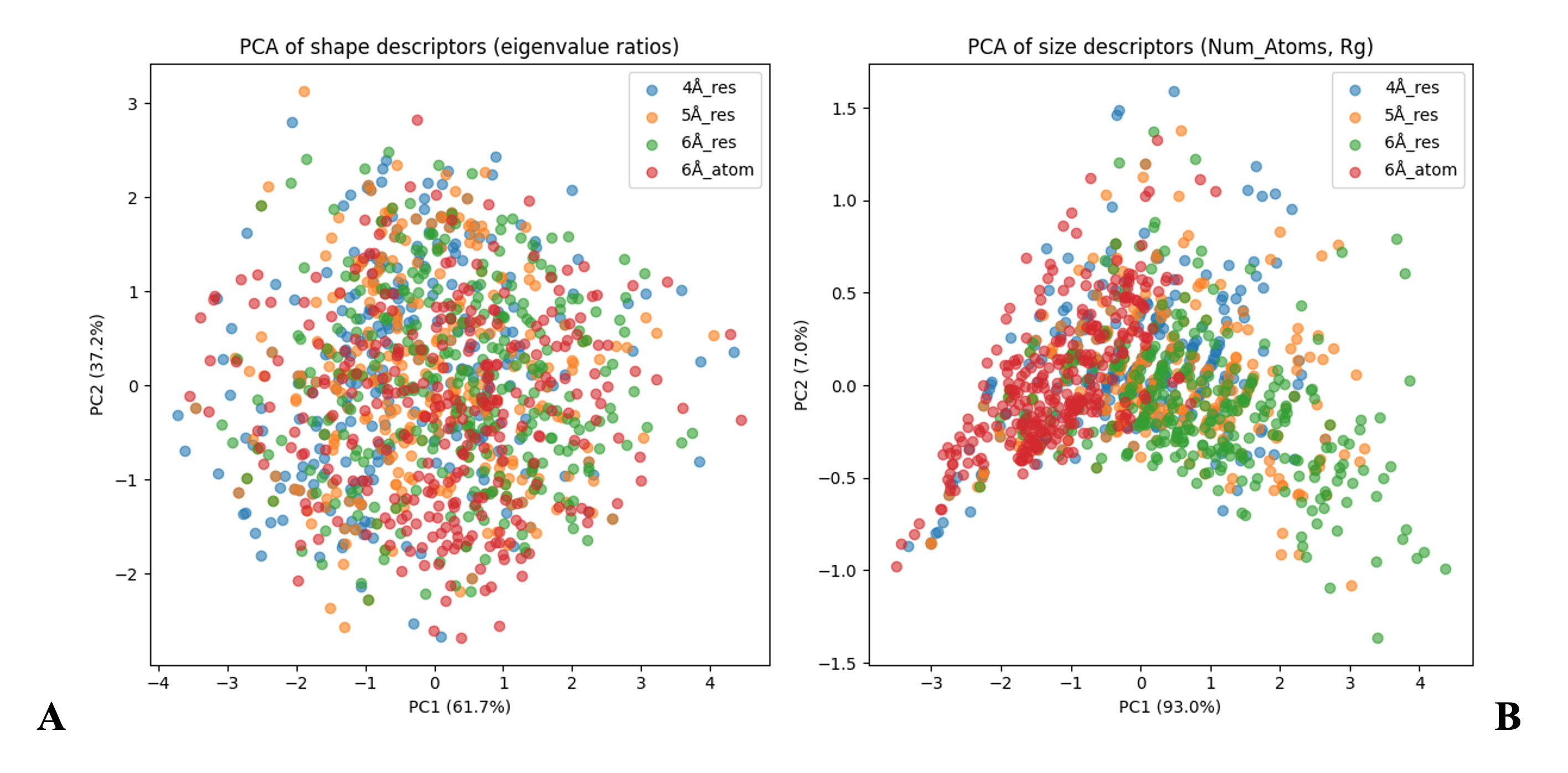

Supplement: Supplementary 1 — Section S1: Table S1 Section S2: Fig. S2 Section S3: Fig. S3 Section S4: Tables S4.2 and S4.3 Section S5: Table S5 Section S6: Tables S6.1 and S6.2 and Fig. S6 Section S7: Fig. S7 and Table S7.2 Section S8: Fig. S8 Section S9: Tables S9.1, S9.2, and S9.3 Section S10: Fig. S10 and Table S10 Data S11: Table S11 [file csbj.0022.f1.zip › FigureS6.jpg]

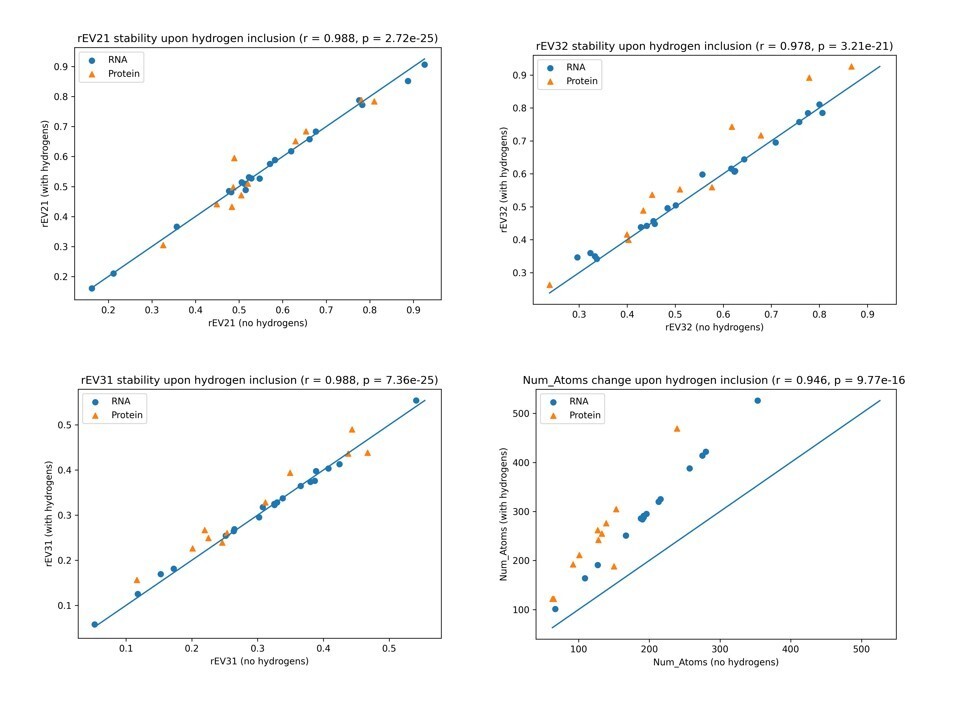

Supplement: Supplementary 1 — Section S1: Table S1 Section S2: Fig. S2 Section S3: Fig. S3 Section S4: Tables S4.2 and S4.3 Section S5: Table S5 Section S6: Tables S6.1 and S6.2 and Fig. S6 Section S7: Fig. S7 and Table S7.2 Section S8: Fig. S8 Section S9: Tables S9.1, S9.2, and S9.3 Section S10: Fig. S10 and Table S10 Data S11: Table S11 [file csbj.0022.f1.zip › FigureS7.jpg]

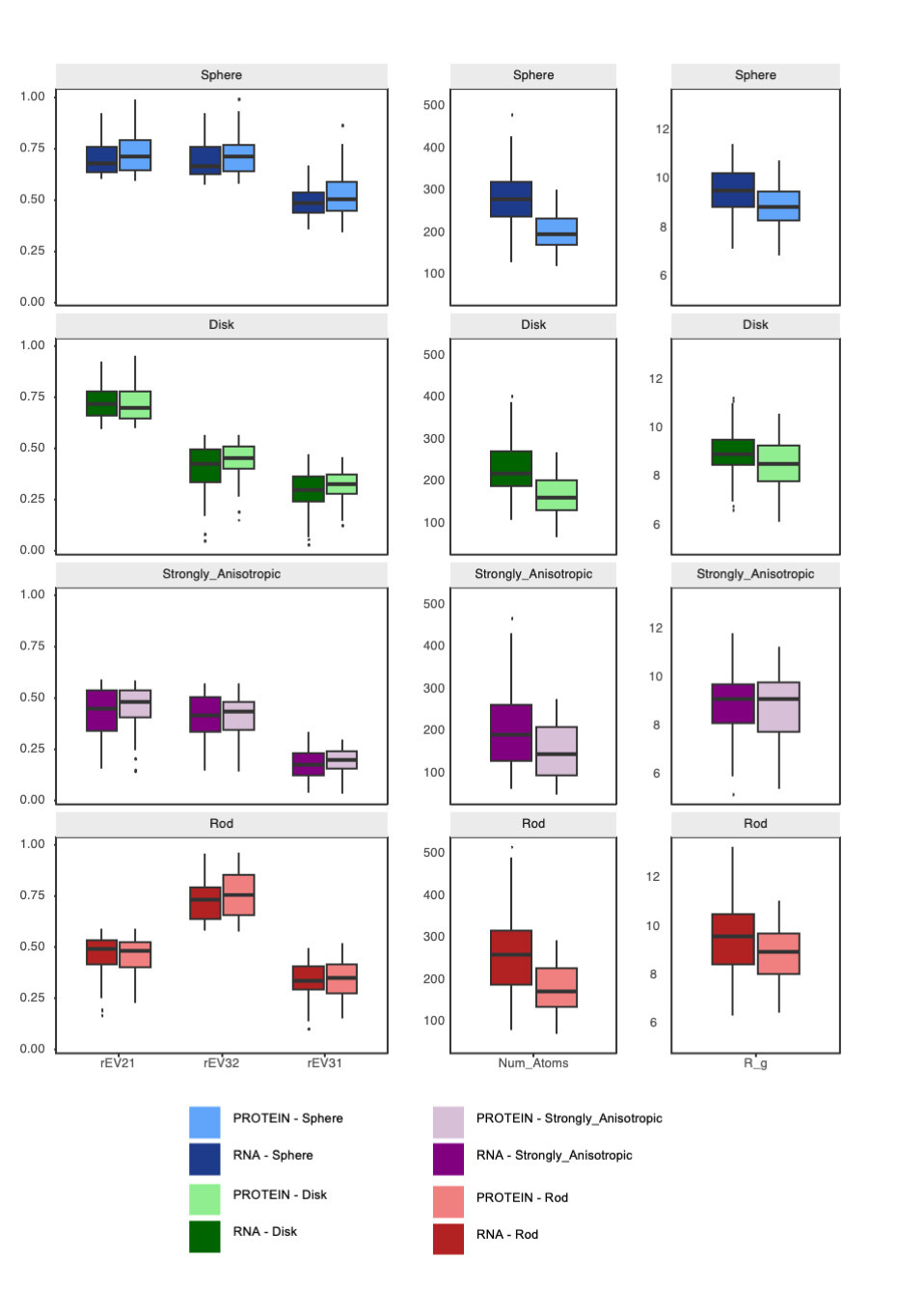

Supplement: Supplementary 1 — Section S1: Table S1 Section S2: Fig. S2 Section S3: Fig. S3 Section S4: Tables S4.2 and S4.3 Section S5: Table S5 Section S6: Tables S6.1 and S6.2 and Fig. S6 Section S7: Fig. S7 and Table S7.2 Section S8: Fig. S8 Section S9: Tables S9.1, S9.2, and S9.3 Section S10: Fig. S10 and Table S10 Data S11: Table S11 [file csbj.0022.f1.zip › FigureS8.jpg]
